# Histone Modifications in Cardiovascular Disease: Mechanisms and Therapeutic Opportunities

**DOI:** 10.1002/mco2.70765

**Published:** 2026-05-13

**Authors:** Yu Zheng, Yu‐Xuan Gao, Mei‐Xing Guo, Li‐Ting Wang, Xiao‐Wen Zheng, Yi‐Hao Liao, Ya Li, Jun‐Ping Zhu, Jia‐Ming Wei

**Affiliations:** ^1^ School of Chinese Medicine Hunan University of Chinese Medicine Changsha China; ^2^ School of Pharmacy Hunan University of Chinese Medicine Changsha China; ^3^ Institute of TCM Diagnostics Hunan University of Chinese Medicine Changsha China

**Keywords:** atherosclerosis, heart failure, histone posttranslational modifications, hypertension, myocardial infarction, myocardial ischemia–reperfusion injury

## Abstract

Cardiovascular diseases remain the leading cause of death worldwide. Maladaptive transcriptional programs drive the fibrosis, hypertrophy, and vascular inflammation that characterize these pathologies. Histone posttranslational modifications regulate these programs by remodeling chromatin accessibility and transcriptional output in cardiomyocytes, vascular cells, and immune cells. These modifications include methylation, acetylation, and metabolite‐derived acylations. While the enzymatic machinery of classical histone marks is increasingly well defined, the cell‐type‐specific integration of these regulators into dynamic cardiovascular networks remains incompletely understood. This narrative review summarizes experimental and human studies published up to early 2026. We examine how classical marks such as H3K27me3 and H3K9ac, alongside emerging metabolic sensors like histone lysine lactylation, shape core pathobiological programs, including oxidative stress responses, endothelial dysfunction, and extracellular matrix remodeling, across major cardiovascular syndromes. We further critically evaluate the enzymatic machinery and pharmacological strategies by contrasting broad‐spectrum histone deacetylase inhibition with precision approaches, including bromodomain inhibition and locus‐selective epigenome editing. Finally, we address translational constraints such as drug delivery and off‐target effects. We propose that single‐cell resolution and spatial multiomics will be essential to identify compartment‐specific targets and advance precision cardiovascular epigenetic therapeutics.

## Graphical abstract

This graphical abstract summarizes how classical and emerging histone posttranslational modifications (PTMs), together with their chromatin regulators (writers, erasers, and readers), govern major pathobiological programs in cardiovascular disease. These programs include inflammation, oxidative and mitochondrial stress, fibrosis and extracellular matrix (ECM) remodeling, endothelial dysfunction, vascular smooth muscle cell (VSMC) phenotypic switching, and cardiac hypertrophy and remodeling. Across heart failure, atherosclerosis, myocardial infarction, myocardial ischemia–reperfusion injury, and hypertension, these shared yet context‐dependent chromatin programs contribute to disease phenotype and therapeutic translation. The figure also outlines the translational landscape, including targetable regulators, major translational constraints, and enabling technologies relevant to precision cardiovascular epigenetic therapeutics. This graphical abstract was created by the authors using BioRender.com. No copyrighted third‐party published images or graphical elements were reproduced. *Abbreviations*: BET, bromodomain and extraterminal domain; ECM, extracellular matrix; HDAC, histone deacetylase; PTMs, posttranslational modifications; VSMC, vascular smooth muscle cell.

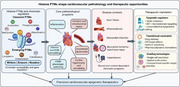

## 1 Introduction

Cardiovascular disease (CVD) comprises a broad spectrum of heart and vascular disorders, including heart failure (HF), atherosclerosis (AS), myocardial infarction (MI), and hypertension. Despite major advances in prevention and treatment, CVD remains the leading cause of global disease burden and death. In the Global Burden of Disease 2023 study, CVD accounted for 19.2 million deaths worldwide and 626 million prevalent cases in 2023 [1]. Yet important forms of residual injury remain despite therapeutic progress. In acute MI, timely reperfusion is essential for myocardial salvage, but the abrupt return of flow can itself trigger additional myocardial injury and necrosis, a phenomenon recognized as myocardial ischemia–reperfusion injury (MIRI) [2, 3].

Beyond inherited susceptibility, cardiovascular phenotypes are also shaped by the dynamic regulation of gene expression in response to environmental and pathological stimuli. Epigenetics refers to heritable changes in gene expression that occur without changes in the DNA sequence. These processes may be influenced by exogenous stimuli and mediate environment–genome interactions, thereby contributing to disease initiation and CVD development [4]. In CVD, the major epigenetic layers include DNA methylation, histone modifications, and RNA‐based mechanisms [4, 5]. Among these, histone modifications modulate gene expression through changes in DNA accessibility and are a key component of chromatin‐based regulation in CVD [4, 6].

Histone posttranslational modifications (PTMs) are increasingly recognized as key regulators of gene expression and disease progression in cardiovascular biology [5]. Chromatin is a dynamic regulatory structure that responds to external cues, and histone modifications are central to this regulation [6]. The repertoire of histone PTMs has also expanded beyond classical methylation and acetylation to metabolite‐linked acylations [6, 7]. Notably, lactate‐derived histone lysine lactylation can directly stimulate gene transcription from chromatin, highlighting a direct link between intermediary metabolism and gene regulation [7]. The dynamic and potentially reversible nature of these modifications makes epigenetic enzymes and chromatin regulators attractive therapeutic targets in CVD [5]. Bromodomain and extraterminal domain (BET) proteins have emerged as acetyl‐lysine readers of therapeutic interest, supported by preclinical HF studies and clinical investigation of apabetalone [5, 8, 9].

Against this background, a focused review of histone modifications in CVD is warranted. In this review, we first summarize the major classes of histone PTMs and the writer–eraser–reader machinery that installs, removes, and interprets these marks. We then integrate mechanistic evidence showing how histone‐dependent chromatin regulation contributes to core pathobiological programs, including inflammation, oxidative and mitochondrial stress, fibrosis and extracellular matrix (ECM) remodeling, vascular smooth muscle cell (VSMC) phenotypic switching, endothelial dysfunction, and cardiac hypertrophy and remodeling. Building on this framework, we next examine disease‐specific evidence across HF, AS, MI, MIRI, and hypertension. We then discuss current therapeutic targeting strategies and the major translational constraints that shape their interpretation and clinical development. Finally, we review emerging technologies and future research directions relevant to precision cardiovascular epigenetic therapeutics.

## 2 Histone Modifications

This section outlines the major classes of histone PTMs and summarizes the principal enzymatic and effector systems that install, remove, or interpret these marks, providing the biochemical basis for the mechanistic and therapeutic sections that follow.

**FIGURE 1:**
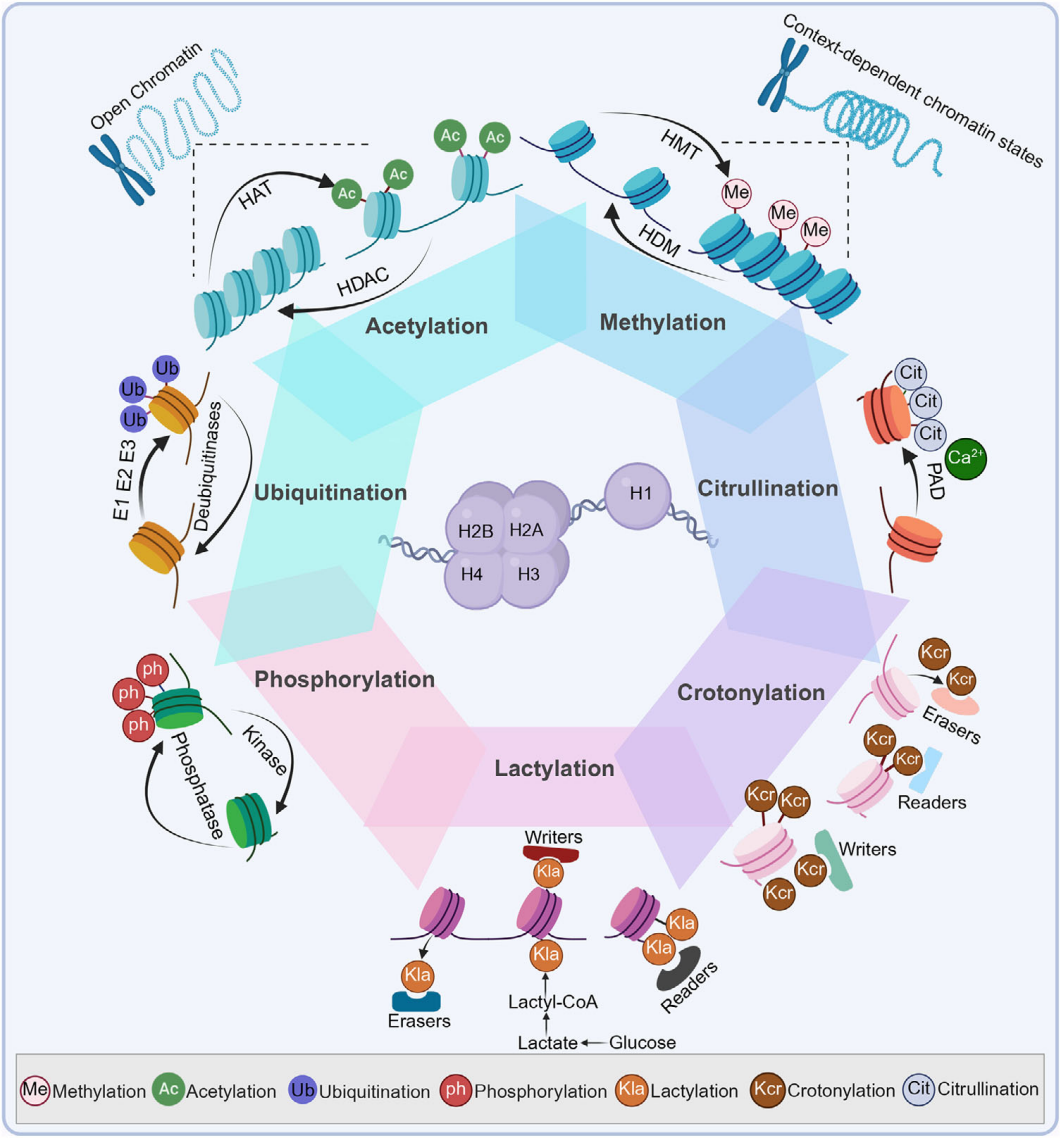
Overview of major histone PTM classes and the enzymatic machinery shaping chromatin states. Schematic overview of the major classes of histone posttranslational modifications (PTMs) and the enzymatic systems that install, remove, or interpret these marks on nucleosomal histones. Classical PTMs include acetylation, regulated by histone acetyltransferases and histone deacetylases, and methylation, regulated by histone methyltransferases and histone demethylases. The figure also summarizes signaling‐responsive phosphorylation, ubiquitination mediated through E1–E2–E3 ligase cascades and reversed by deubiquitinases, and calcium‐dependent citrullination catalyzed by peptidylarginine deiminases. Emerging metabolite‐linked lysine acylations, including lactylation and crotonylation, are shown as additional layers linking intermediary metabolism to chromatin regulation. Together, these PTM systems shape chromatin accessibility and context‐dependent transcriptional states. Ac, acetylation; Cit, citrullination; HAT, histone acetyltransferase; HDAC, histone deacetylase; HDM, histone demethylase; HMT, histone methyltransferase; Kcr, lysine crotonylation; Kla, lysine lactylation; Me, methylation; PAD, peptidylarginine deiminase; ph, phosphorylation; Ub, ubiquitination. Created by the authors using BioRender.com; no copyrighted third‐party published images were reproduced.

Histones are basic proteins in eukaryotic chromatin and include core histones (H2A, H2B, H3, H4) and linker histones (H1). Core histones combine with DNA to form nucleosome core particles, while linker histones bind to nucleosomes to form chromatosomes and contribute to higher‐order chromatin compaction. PTMs of histones dynamically regulate chromatin accessibility and orchestrate gene expression and DNA metabolism [10]. Histone modifications refer to chemical alterations of histone amino acid residues, including methylation, acetylation, phosphorylation, glycosylation, and ubiquitination. These marks regulate chromatin compaction and DNA accessibility by altering histone charge and/or recruiting specific regulatory proteins, thereby modulating transcription [11]. For example, the addition or removal of methyl, acetyl, or phosphate groups can change transcription factor access and lead to altered gene expression [12]. Current evidence indicates that histone modifications exert significant effects in the cardiovascular system and are also implicated in non‐CVDs, including glioma and ovarian cancer [13, 14, 15]. In the cardiovascular system, histone modifications regulate cardiac fibroblast activation and cardiomyocyte hypertrophy, mediate chromatin remodeling, and reprogram stress‐induced and profibrotic gene expression. These processes promote cardiac fibrosis and hypertrophy, thereby driving the development and progression of CVD [8, 16] (Figure 1).

### 2.1 Histone Methylation

Histone methylation is a key epigenetic modification that primarily occurs at lysine (Lys/K) and arginine (Arg/R) residues of histones H3 and H4 at specific sites. In H3, methylation can occur at lysines 4, 9, 26, 27, 36, 56, and 79, and arginines 2, 8, and 17; in H4, at lysines 5, 12, and 20, and arginine 3 [17]. Methyltransferases (“writers”) catalyze the addition of methyl groups, generating monomethylated (me1), dimethylated (me2), or trimethylated (me3) states, with biological effects determined by the modified residue and methylation degree [17]. For example, H3K4me3 and H3K36me3 are generally associated with transcriptional activation, whereas H3K9me3, H3K27me3, and H4K20me3 are generally associated with transcriptional repression [18]. Methylated residues can also be recognized by reader proteins or block other chromatin‐associated proteins, influencing downstream processes [11, 19].

Histone methylation is a dynamic and reversible process maintained through the coordinated actions of histone methyltransferases (HMTs) and histone demethylases (HDMs), which regulate lysine or arginine methylation to preserve genome stability and cellular homeostasis [17]. HMTs include lysine methyltransferases (KMTs) and protein arginine methyltransferases (PRMTs), with KMTs further classified into Su(var)3‐9, enhancer of zeste, and trithorax (SET) domain‐containing enzymes and non‐SET domain enzymes [17]. HDMs are grouped into two main families according to catalytic mechanism. These comprise lysine‐specific demethylases (LSD, also termed the KDM1 family) and Jumonji C (JmjC) domain‐containing lysine demethylases (KDMs) [17]. The LSD family, comprising LSD1 (KDM1A) and LSD2 (KDM1B), removes mono‐ and dimethyl groups from lysine residues via a flavin adenine dinucleotide‐dependent oxidative reaction [20]. JmjC demethylases are Fe^2^^+^/α‐ketoglutarate‐dependent and encompass seven evolutionarily distinct subfamilies, capable of removing mono‐, di‐, and trimethyl marks from lysine residues; arginine methylation exists as monomethylarginine, asymmetric dimethylarginine, and symmetric dimethylarginine [17, 19]. These enzyme classes enable precise and reversible regulation of histone methylation.

### 2.2 Histone Acetylation

Histone acetylation, a common PTM, occurs on lysine residues across the core histones (H2A, H2B, H3, and H4) [21]. It is dynamically regulated by histone acetyltransferases (HATs) and histone deacetylases (HDACs) [6]. HATs transfer an acetyl group from acetyl coenzyme A (acetyl‐CoA) to the ε‐amino group of lysine, neutralizing the histone's positive charge, weakening histone–DNA interactions, relaxing chromatin, and promoting transcription factor binding [6]. Classically, type A HATs are nuclear and involved in transcriptional activation, whereas type B HATs are cytoplasmic and acetylate newly synthesized histones [22]. HATs are commonly grouped into three major groups. These comprise the GCN5‐related N‐acetyltransferase family, the MOZ Ybf2 Sas3 Sas2 Tip60 (MYST) family, and the p300/CREB‐binding protein (p300/CBP) group [6]. HDACs remove lysine acetyl groups, restore positive charge, tighten histone–DNA contacts, condense chromatin, and repress transcription; many also act on nonhistone substrates. HDACs are grouped into four classes. Class I comprises HDAC1, HDAC2, HDAC3, and HDAC8 and is chiefly nuclear. Class II subdivides into IIa with HDAC4, HDAC5, HDAC7, and HDAC9, and IIb with HDAC6 and HDAC10. Class III comprises the sirtuins (SIRT1–SIRT7). Class IV contains HDAC11. Classes I, II, and IV are zinc dependent, whereas Class III is nicotinamide adenine dinucleotide (NAD^+^) dependent [23].

### 2.3 Histone Phosphorylation

Histone phosphorylation is a widespread PTM in eukaryotes that enables rapid cellular responses to environmental stimuli [24]. It involves the addition of a phosphate group to serine, threonine, or tyrosine residues, primarily on histone tails [25, 26] and is dynamically regulated by protein kinases and phosphatases [26]. Kinases transfer the γ‐phosphate group of adenosine 5′‐triphosphate (ATP) to histone residues, generating phosphorylation marks such as H2AX Ser139 phosphorylation (γH2AX) and histone H3 Ser10 phosphorylation (H3S10ph) [26, 27]. Phosphatases remove these groups to reverse the modification [26, 28]. Phosphorylated residues act as molecular “switches” recognized by phospho‐binding modules that recruit downstream effector proteins [26, 29]. Phosphorylation coordinates chromatin remodeling by modulating chromatin‐associated interactions and can influence neighboring modifications such as methylation and acetylation [30]. Crosstalk with other modifications underlies diverse processes. H3S10ph disrupts HP1 binding to methylated H3K9 and is functionally linked to GCN5‐mediated histone H3 lysine 14 acetylation (H3K14ac) during transcriptional activation [26, 30]; histone H2B Ser36 phosphorylation controls adipogenesis [31]; and H1.4 phosphorylation is associated with transcription activation [32]. These events collectively regulate key nuclear functions, including DNA damage repair and chromosome condensation [26, 30].

### 2.4 Histone Lactylation

Histone lysine lactylation (Kla) is a recently identified epigenetic modification derived from lactate metabolism, and p300 can catalyze lysine lactylation using L‐lactyl coenzyme A in vitro [7]. Under conditions of enhanced glycolysis, including hypoxia and bacterial challenge, intracellular lactate increases and Kla rises; 28 lactylation sites have been identified on core histones in human and mouse cells. Kla shows temporal dynamics distinct from histone acetylation. In bacterially challenged M1 macrophages, lactate accumulation promotes H3K18 lactylation during the late phase of activation, a mark associated with the induction of homeostatic and wound healing genes and a shift toward a reparative transcriptional program. This pattern has been framed as an endogenous “lactate clock” linking metabolic state to transcriptional control, and Kla has been discussed in broader pathophysiological contexts, including infection and cancer [7].

### 2.5 Histone Ubiquitination

Histone ubiquitination is a chromatin signal that regulates transcription and coordinates the DNA damage response (DDR). Ubiquitin, a 76‐amino‐acid protein, is conjugated to histone lysines through the conserved E1–E2–E3 cascade, in which an ATP‐dependent E1 forms a thioester with ubiquitin, an E2 receives and carries ubiquitin, and an E3 catalyzes isopeptide bond formation; deubiquitinating enzymes remove the mark and restore the unmodified histone [33]. Monoubiquitination of core histones, most prominently H2A at K13/K15 and K127/K129 and H2B at K120, does not signal degradation but alters nucleosome dynamics and promotes crosstalk with other histone marks; in the DDR, H2A ubiquitination also creates binding platforms for repair factors such as 53BP1 and BARD1/BRCA1 [33, 34]. Linkage type confers distinct outcomes, with K48‐linked polyubiquitin serving as the canonical proteasomal degradation signal, whereas K63‐linked chains function in nonproteolytic signaling, including extension of monoubiquitin marks and scaffolding of repair‐complex assembly [33, 35].

### 2.6 Histone Crotonylation

Histone crotonylation (Kcr) is a recently identified epigenetic modification in which crotonyl‐CoA donates a crotonyl group to the ε‐amino group of lysine residues in histones [36, 37]. It is predominantly enriched in transcriptionally active regions, such as promoters and enhancers. Common modification sites include histone H3 (K9, K18, K23) and H4 (K5, K8, K12) [36]. As a short‐chain lysyl acylation, Kcr shares sites and enzymes with acetylation, since p300/CBP can use crotonyl‐CoA to install Kcr; intracellular crotonyl‐CoA levels bias the balance between crotonylation and acetylation at overlapping lysines [37]. The crotonyl group's rigid, planar four‐carbon chain increases hydrophobic contacts and is selectively read by YEATS domains via π‐aromatic stacking [38]. Histone Kcr has been linked to postmeiotic male germ cell gene expression and activation of sex chromosome‐linked genes during spermatogenesis [39]. In kidney‐injury models, it also links metabolic cues to transcriptional programs involving peroxisome proliferator‐activated receptor‐γ coactivator‐1α and sirtuin 3 (SIRT3) [40]. Dysregulation of Kcr has been associated with hepatocellular carcinoma [41] and latent human immunodeficiency virus infection [42] and has also been implicated in HSV‐1 infection [43] and MIRI [44].

### 2.7 Histone Citrullination

Histone citrullination is a Ca^2+^‐dependent deimination catalyzed by the peptidylarginine deiminase (PAD) family, converting positively charged arginine residues to neutral citrulline with release of ammonia (NH3) [45, 46]; PAD4 can also demethyliminate methyl‐Arg to produce methylamine [47]. Key reported histone sites include H1 R54, H3 R2, R8, R17, and R26, and H4 R3 [46, 47, 48, 49]. Citrullination neutralizes the positive charge on histones, weakens histone–DNA electrostatic interactions, drives chromatin decondensation, and facilitates the recruitment of SWItch/sucrose nonfermentable complexes and RNA polymerase II (Pol II) [46]. In addition, it modulates transcription through crosstalk with arginine‐ and lysine‐methylation. PAD4 removes methyl marks at H3R17 to antagonize PRMT4, whereas PAD2‐mediated H3R26 citrullination blocks polycomb repressive complex 2 (PRC2)‐mediated H3K27 methylation, thereby regulating estrogen receptor target gene transcription [47, 48]. Physiologically, PAD1‐mediated citrullination of H3R2/8/17 and H4R3 is essential for early embryonic genome activation [49]; in neutrophils, PAD4 promotes histone citrullination and chromatin decondensation to drive neutrophil extracellular trap (NET) formation [50]; and PAD2‐dependent citrullination supports wound responses and tissue regeneration in zebrafish [51]. Pathologically, heightened PAD4 activity enhances NET formation (NETosis) and exacerbates inflammation, while NETs promote metastatic progression after surgical stress and activate TLR9‐dependent signaling in cancer cells, partly through released HMGB1 [50, 52].

Overall, histone PTMs regulate chromatin through several recurring biochemical modes, including covalent mark installation and removal, crosstalk between neighboring modifications, and the recruitment of effector proteins. The mechanistic framework is most fully defined for methylation and acetylation, whereas emerging acylations and citrullination remain less completely resolved. Their phenotypic consequences depend on genomic locus, cell type, and stimulus context, helping explain why the same chromatin regulator can be protective in one setting yet maladaptive in another. Having outlined the major classes of histone PTMs, we next integrate mechanistic evidence showing how these marks contribute to key pathobiological programs that drive cardiovascular remodeling.

## 3 Mechanistic Insights: How Histone Modifications Regulate Cardiovascular Pathophysiology

**FIGURE 2:**
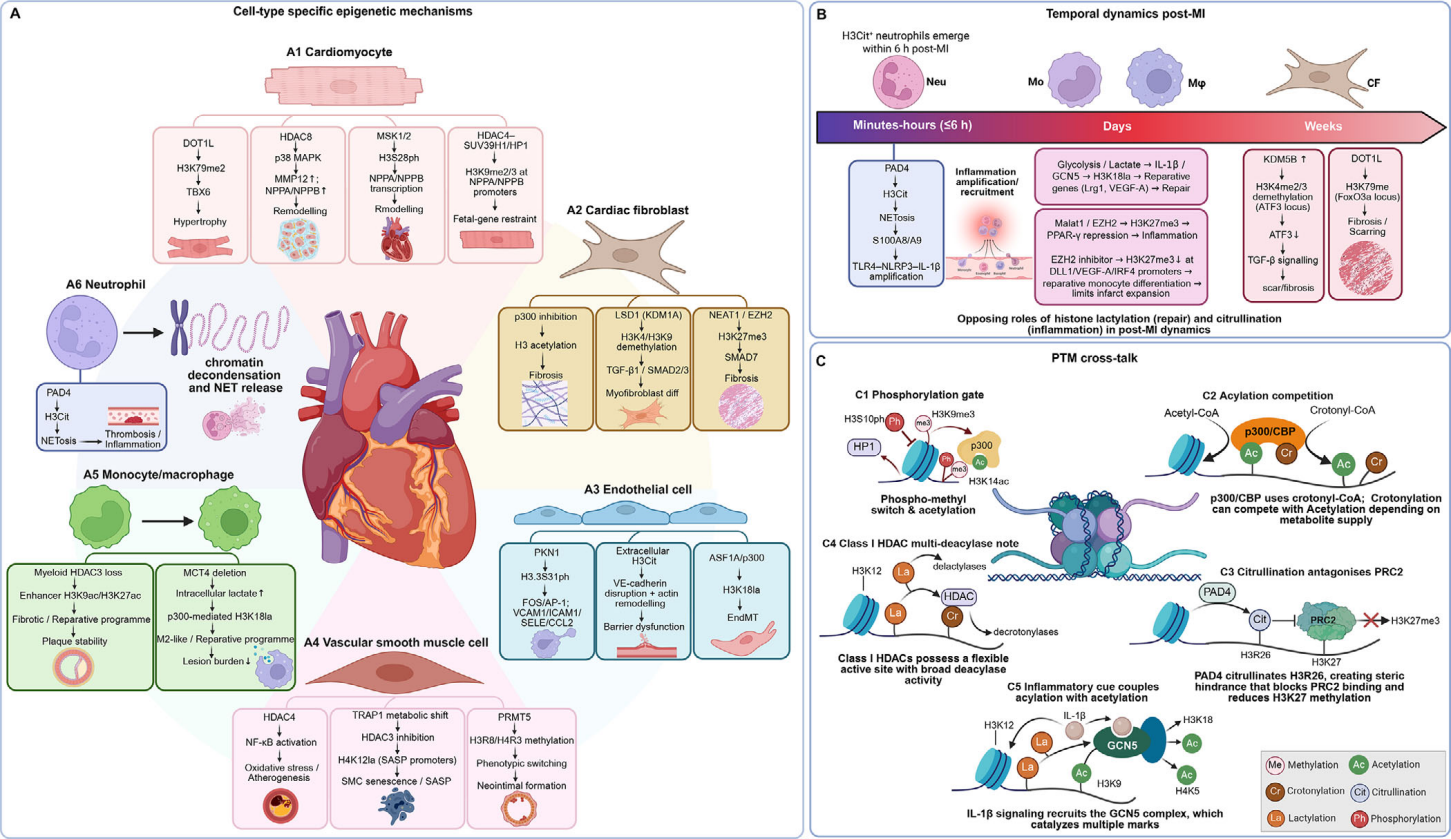
Histone PTM programs across cardiovascular‐relevant cell types, post‐MI temporal progression, and PTM cross‐talk. (A) Representative histone marks and their regulators across cardiomyocytes, cardiac fibroblasts, endothelial cells, vascular smooth muscle cells, monocytes/macrophages, and neutrophils, together with the associated phenotypic outputs. (B) Selected PTM programs aligned with the post‐MI timeline, showing the transition from early PAD4–H3Cit–NETosis‐driven inflammatory amplification to later methylation‐ and lactylation‐linked reparative and fibrotic remodeling. (C) Representative modes of PTM cross‐talk, including the phospho‐methyl switch, competition between acetylation and crotonylation, PAD4‐mediated antagonism of PRC2, inflammatory cue‐coupled lactylation and acetylation, and the broad multideacylase activity of class I HDACs. Arrows indicate activation or induction, and blunt‐ended lines indicate repression or inhibition. AP‐1, activator protein 1; ASF1A, antisilencing function 1A; CBP, CREB‐binding protein; CCL2, C–C motif chemokine ligand 2; CF, cardiac fibroblast; DLL1, delta‐like canonical Notch ligand 1; DOT1L, disruptor of telomeric silencing 1‐like; EndMT, endothelial‐to‐mesenchymal transition; EZH2, enhancer of zeste homolog 2; FOS, Fos proto‐oncogene, AP‐1 transcription factor subunit; FoxO3a, forkhead box O3a; GCN5, general control nonderepressible 5; H3Cit, citrullinated histone H3; HDAC, histone deacetylase; HP1, heterochromatin protein 1; ICAM1, intercellular adhesion molecule 1; IL, interleukin; IRF4, interferon regulatory factor 4; KDM, lysine demethylase; LSD1, lysine‐specific demethylase 1; LRG1, leucine‐rich alpha‐2‐glycoprotein 1; Malat1, metastasis‐associated lung adenocarcinoma transcript 1; MAPK, mitogen‐activated protein kinase; MCT4, monocarboxylate transporter 4; MI, myocardial infarction; Mo, monocyte; MMP12, matrix metallopeptidase 12; MSK1/2, mitogen‐ and stress‐activated protein kinase 1/2; Mφ, macrophage; NEAT1, nuclear paraspeckle assembly transcript 1; NET, neutrophil extracellular trap; Neu, neutrophil; NF‐κB, nuclear factor kappa B; NLRP3, NLR family pyrin domain containing 3; NPPA, natriuretic peptide A; NPPB, natriuretic peptide B; PAD4, peptidylarginine deiminase 4; PKN1, protein kinase N1; PPAR‐γ, peroxisome proliferator‐activated receptor gamma; PRC2, polycomb repressive complex 2; PRMT5, protein arginine methyltransferase 5; PTM, posttranslational modification; SASP, senescence‐associated secretory phenotype; SELE, E‐selectin; SMAD, SMAD family member; SUV39H1, suppressor of variegation 3–9 homolog 1; TBX6, T‐box transcription factor 6; TGF‐β, transforming growth factor beta; TLR4, Toll‐like receptor 4; TRAP1, TNF receptor‐associated protein 1; VCAM1, vascular cell adhesion molecule 1; VE‐cadherin, vascular endothelial cadherin; VEGF‐A, vascular endothelial growth factor A; VSMC, vascular smooth muscle cell. This figure was created by the authors with BioRender.com and does not reproduce any copyrighted third‐party published material.

Histone PTMs typically fine‐tune the magnitude and duration of transcriptional programs rather than acting as binary switches. Their phenotypic effects reflect how combinations of marks are written, erased, and read across cell types and disease stages. Here, we organize the literature around major injury‐associated processes, including sterile inflammation, oxidative and mitochondrial stress, fibrosis and ECM remodeling, VSMC phenotypic switching, endothelial dysfunction, and hypertrophic remodeling. This framework helps interpret disease‐specific mechanisms across major cardiovascular syndromes (Figure 2).

### 3.1 Regulation of Inflammation

Inflammatory signaling contributes to multiple stages of CVD, from early endothelial activation in AS to later vascular and myocardial remodeling [5, 53]. Histone PTMs modulate how vascular and cardiac cells respond to inflammatory and stress cues [5, 53, 54]. Histone acetylation and methylation, together with emerging PTMs such as lactylation and arginine citrullination, regulate chromatin accessibility and inflammatory gene expression in response to proinflammatory cytokines and related signaling pathways [53, 54]. In experimental models, particularly AS and MI, epigenetic and NET‐associated pathways are accompanied by differences in cytokine output, leukocyte recruitment, NET formation, and vascular or myocardial remodeling [5, 53, 55, 56].

Class I HDACs influence nuclear factor κB (NF‐κB) and signal transducer and activator of transcription signaling and thereby modulate inflammatory gene expression in cellular models [57, 58]. For example, in macrophages, HDAC3 promotes NLR family pyrin domain‐containing 3 (NLRP3)‐dependent caspase‐1 maturation and interleukin‐1β (IL‐1β) production [59]; in endothelial cells (ECs), HDAC3 supports tumor necrosis factor‐α (TNF‐α)‐induced vascular cell adhesion molecule 1 (VCAM1) expression and monocyte adhesion [60]. Endothelial HDAC3 is also required for survival responses to disturbed flow and contributes to AS development [61]. Strikingly, macrophage HDAC3 deficiency shifts lesion composition toward a more stable phenotype, with a thicker fibrous cap and no difference in necrotic core size [62]. In VSMCs, HDAC4 amplifies TNF‐α–driven inflammatory responses by enhancing NF‐κB activity and oxidative stress [63]. HDAC7 couples lipopolysaccharide (LPS)/TLR4 signaling to glycolysis via deacetylation of pyruvate kinase M2, promoting HIF‐1α activity and sustaining IL‐1β and chemokine expression [64]. Among Class III deacetylases, SIRT1 restrains T cell activation [65]. In cardiometabolic disease models, macrophage SIRT3 deficiency is associated with exacerbated chronic low‐grade inflammation and metabolic dysfunction [66]. During NF‐κB signaling, CBP/p300 acts as a HAT coactivator at the IL‐6 promoter, and its acetyltransferase activity is required for maximal induction [67]. Similarly, p300 acetyltransferase activity is required for proinflammatory mediator‐driven activation of the cyclooxygenase‐2 promoter [68]. In human macrophages, inflammatory stimulation increases p300 and HAT1 enrichment at the NADPH oxidase 5 (NOX5) promoter with enhanced local histone acetylation and NOX5 upregulation, supporting an HAT‐dependent route to oxidative inflammatory output [69]. Beyond acetylation, histone methylation and demethylation regulate the magnitude and persistence of inflammatory gene activation. Histone lysine demethylases remove activating or repressive methyl marks, including activating histone H3 lysine 4 di‐ and trimethylation (H3K4me2/3) and repressive H3K27me3, at promoters and enhancers of innate immune genes. This regulation modulates TLR signaling and Type I interferon (IFN‐I) responsive transcription and affects cytokine production, including IL‐6. In some models, demethylase‐dependent pathways engage autophagy to limit NLRP3 inflammasome activation and dampen inflammatory injury [70, 71, 72]. In CVD models, both HMTs and demethylases have been linked to vascular inflammation and AS [53]. The H3K9 methyltransferase SET domain bifurcated 2 reinforces repression of IFN‐I response genes and inflammatory cytokine and chemokine genes in hematopoietic cells, and its loss in hyperlipidemic mice increases vascular inflammation and accelerates atherosclerotic plaque growth [73]. Conversely, pharmacological inhibition of KDM1A reduces inflammatory marker expression and lesion burden in AS models [74].

Kla was first described in macrophages as a glycolysis‐responsive modification that accumulates on H3 and other lysine residues during late M1 polarization, where it activates a set of wound‐healing and homeostatic genes and favors the transition from a proinflammatory to a reparative phenotype [7]. Subsequent studies in macrophages and cardiovascular injury models show that histone lactylation supports the transition from inflammatory toward reparative gene programs [75, 76]. After MI, lactate‐driven H3K18la in monocytes and macrophages links metabolic state to induction of reparative gene programs [76]. This mechanism is associated with proangiogenic and inflammation‐resolving transcription during postinfarction repair [76].

Arginine citrullination mediated by PADs also participates in the control of inflammatory responses. In neutrophils, PAD4‐dependent histone citrullination promotes chromatin decondensation and is required for NET formation [77, 78]. In CVD, NET structures containing citrullinated histones have been detected in atherosclerotic plaques, and pharmacological inhibition of PAD activity with the pan‐PAD inhibitor Cl‐amidine reduces atherosclerotic lesion burden and delays arterial thrombosis in apolipoprotein E‐deficient (ApoE^−^/^−^) mice [55]. Experimental AS studies identify citrullinated histone H3 (H3Cit)‐positive NETs in plaques and link persistent NETs to plaque inflammation and impaired resolution [79]. In ST‐elevation MI (STEMI) cohorts, including patients treated during percutaneous coronary intervention, circulating H3Cit‐based NET markers associate with cardiac magnetic resonance defined myocardial salvage and infarct size, and higher H3Cit–DNA levels predict 1‐year major adverse cardiovascular events (MACE) and mortality [80, 81].

Histone PTMs are recurrent regulators of inflammatory signaling in cardiovascular contexts [5, 53]. Reversible lysine acetylation and methylation regulate inflammatory gene expression in vascular and innate immune settings [5, 53]. Metabolite‐linked acylation, particularly histone lactylation, favors reparative and inflammation‐resolving gene programs, whereas PAD4‐dependent histone citrullination within NET‐associated pathways further shapes thromboinflammatory outputs [7, 55, 56, 76, 82].

### 3.2 Oxidative Stress and Mitochondrial Dysfunction

Oxidative stress and mitochondrial dysfunction are tightly coupled in CVD and injury. Redox adaptation is shaped by acetylation‐linked regulators and by metabolite‐derived lysine acylations on histone and nonhistone substrates.

SIRTs regulate cardiovascular redox homeostasis. Mitochondrial SIRT3 deacetylates and activates manganese superoxide dismutase (SOD) and isocitrate dehydrogenase 2 (IDH2) to maintain mitochondrial redox balance [83, 84]. Consistent with this role, SIRT3 deletion is associated with impaired respiration, increased H_2_O_2_, and cardiac hypertrophy and fibrosis with oxidative stress [85]. SIRT1 also shows a gene‐dose effect in the heart. Low‐to‐moderate cardiac overexpression limits age‐related hypertrophy and fibrosis and increases antioxidant expression, including catalase, whereas high or constitutive higher‐dose overexpression disrupts mitochondrial homeostasis and leads to cardiomyopathy [86, 87]. In the arterial wall, SIRT6 haploinsufficiency increases superoxide and impairs endothelium‐dependent relaxation; NAD(P)H oxidase inhibition with apocynin reduces superoxide and improves endothelial function, consistent with NAD(P)H oxidase‐derived oxidative stress in the arterial wall [88].

Among Class I/II deacetylases, cytoplasmic HDAC6 modulates redox signaling through nonhistone substrates. In diabetic myocardial ischemia–reperfusion (MI/R), HDAC6 inhibition increases peroxiredoxin 1 acetylation and reduces oxidative injury [89]. In doxorubicin (DOX)‐treated human cardiomyocytes, HDAC6 upregulation parallels increased NOX2 and reactive oxygen species (ROS), whereas indole‐3‐propionic acid‐mediated suppression of HDAC6 reduces NOX2, lowers oxidative stress, and improves viability; HDAC6 overexpression reverses these effects [90]. p300 provides a complementary acetylation‐linked entry point into mitochondrial regulation. In the postnatal heart, it supports expression of nuclear genes encoding mitochondrial proteins, and dominant‐negative p300 impairs mitochondrial gene expression, lowers ATP, and causes severe cardiomyopathy [91]. Thus, acetylation shapes mitochondrial energetics and antioxidant defense, whereas NOX‐derived ROS is directly linked to the SIRT6 and HDAC6 pathways discussed above.

KMTs and demethylases regulate redox pathways in vascular lesions and during myocardial injury. In AS, the H3K4 methyltransferase SET7 is upregulated in atherosclerotic tissue and proinflammatory macrophages and promotes NOX gene expression [92]. In the atherosclerotic aorta of ApoE^−^/^−^ mice, H3K4me1 and SET7 are increased, and (R)‐PFI‐2 lowers aortic NOX1/2/4 and NOX subunit p22phox messenger RNA (mRNA) and protein levels, reduces 3‐nitrotyrosine and 4‐hydroxynonenal (4‐HNE) adduct formation, and attenuates NLRP3/Casp1 induction and downstream inflammasome activation [92]. In ApoE^−^/^−^ mice, KDM1A inhibition similarly decreases aortic NOX1/2/4 and p22phox expression and reduces 4‐HNE adducts; in HEK293 reporter cells, KDM1A overexpression increases NOX1/2/4/5 and p22phox transcripts, consistent with a KDM1A‐dependent epigenetic program that favors NOX upregulation and oxidative stress [74]. Plant homeodomain finger protein 8 (PHF8) shows the opposite direction in the injured myocardium. PHF8 increases forkhead box A2 (FOXA2) expression in a HDM‐dependent manner and reduces repressive histone marks at the FOXA2 promoter; PHF8 overexpression in left anterior descending coronary artery ligation models and oxygen–glucose deprivation/reoxygenation cardiomyocytes reduces ROS, restores SOD and catalase activity, and limits apoptosis, with cardiomyocyte data supporting FOXA2 as a downstream effector [93]. In AS, methylation programs can reinforce NOX‐associated oxidative stress, whereas PHF8–FOXA2 signaling supports antioxidant defense in myocardial injury models [74, 92, 93].

In ischemic myocardium, protein lysine crotonylation is induced predominantly on mitochondrial and sarcomeric proteins [44]. In a mouse ischemia–reperfusion (I/R) model, sodium crotonate improves left ventricular (LV) function and reduces fibrosis and apoptosis [44]. Site‐specific lysine crotonylation of IDH3A at Lys199 suppresses BCL2/adenovirus E1B 19 kDa‐interacting protein 3‐dependent mitophagy and apoptosis, whereas crotonylation of tropomyosin 1 at Lys28/29 improves cytoskeletal organization and limits cardiomyocyte apoptosis [44].

Lactate‐driven Kla connects glycolytic remodeling to redox‐linked chromatin regulation in cardiovascular contexts. In hypoxic cardiomyocytes, enhanced glycolysis and lactate accumulation increase Kla, activate Wnt/β‐catenin signaling, and promote ferroptosis with ROS accumulation, iron overload, and lipid peroxidation [94]. In MI/R, cardiomyocyte histone H3 lactylation has been linked to preservation of aerobic glycolysis and reduced cardiomyocyte death [95]. In hypoxic pulmonary hypertension models, mROS‐driven glycolytic remodeling increases H3K18la at HIF‐1α target promoters and sustains proliferative programs in pulmonary artery smooth muscle cells [96].

Acetylation‐ and methylation‐linked chromatin programs, together with metabolite‐derived lysine acylation pathways such as crotonylation on mitochondrial and contractile proteins and Kla, contribute to the regulation of oxidative stress and mitochondrial function in CVD. In this setting, histone‐regulatory enzymes and metabolite‐linked acylation pathways couple redox imbalance to mitochondrial and transcriptional remodeling.

### 3.3 Fibrosis and ECM Remodeling

Fibrosis in the heart involves excessive accumulation of ECM proteins, particularly Collagens I and III, which stiffen the ventricular wall, reduce compliance, and impair electrical conduction, thereby promoting progression to HF [97, 98]. Cardiac fibroblasts are the principal source of ECM in the myocardium [99, 100]. In response to pressure overload, metabolic stress, or ischemic injury, cardiac fibroblasts become activated and differentiate into proliferative, α‐smooth muscle actin (α‐SMA)‐positive myofibroblasts that secrete large amounts of collagens and other matrix components, thereby driving interstitial fibrosis [98, 101, 102].

Histone acetylation is a central regulator of cardiac fibroblast activation and ECM deposition [99]. In fibroblasts, transforming growth factor‐β (TGF‐β) increases Type I collagen transcription by recruiting the HAT p300 to SMAD2/3 complexes at the collagen Type I alpha 2 chain (COL1A2) promoter, thereby enhancing local histone H4 acetylation and promoting COL1A2 transcription [103]. In Angiotensin II (Ang II)‐driven hypertension models, profibrotic cues increase p300‐dependent histone H3K9 acetylation in hypertensive myocardium, promote myofibroblast differentiation, and increase collagen production [104].

Inhibitors of p300, including L002 and C646, reduce histone H3K9 acetylation, limit myofibroblast accumulation, and lessen interstitial or perivascular fibrosis in these settings [104]. Curcumin inhibits nuclear acetylation and GATA4 acetylation in cultured cardiomyocytes and reduces perivascular fibrosis in vivo, whereas GO‐Y030 inhibits histone H3K9 acetylation and suppresses perivascular and LV fibrosis after pressure overload [105, 106]. These studies identify p300‐dependent histone acetylation as a key epigenetic axis driving profibrotic transcription and ECM remodeling [103, 104]. Class I and IIa HDACs reinforce profibrotic gene programs in cardiac fibroblasts and failing hearts [99, 100, 107]. Across pressure overload, hypertension, and MI models, pharmacological HDAC inhibition reduces collagen accumulation and fibrotic remodeling and improves diastolic or systolic function. Crucially, in a post‐MI HF model, selective Class I inhibition with mocetinostat also suppresses activation of CD90^+^ cardiac fibroblasts [108].

Histone methylation is an important regulator of fibrotic gene expression in the injured heart [109, 110, 111]. In Ang II‐driven pathological hypertrophy models, the H3K4 methyltransferase SET1 is recruited to the endothelin‐1 promoter in ECs and increases H3K4 trimethylation and endothelin‐1 transcription [109]. Endothelial SET1 depletion attenuates Ang II‐induced cardiac fibrosis [109]. In pressure‐overload models, cardiomyocyte KDM3A reduces repressive H3K9me2 at the TIMP1 promoter; loss of KDM3A suppresses TIMP1 and other ECM‐related genes and reduces matrix accumulation [110]. In atrial fibrillation, the H3K27 methyltransferase enhancer of zeste homolog 2 (EZH2) is upregulated in atrial tissue and fibroblasts and promotes fibroblast differentiation and ECM production through the Ang II–TGF‐β–Smad signaling pathway [111]. Both genetic knockdown and pharmacological inhibition of EZH2 reduce α‐SMA and collagen expression and thereby attenuate atrial fibrosis [111]. SET1‐mediated H3K4 trimethylation, KDM3A‐regulated H3K9me2, and EZH2‐associated H3K27 methylation converge on fibrogenic transcriptional control in endothelial, ventricular, and atrial settings [109, 110, 111].

Additional PTMs beyond acetylation and methylation have been implicated in cardiac remodeling and fibrosis‐related programs in CVD [112, 113]. In leptin receptor‐deficient (db/db) mice subjected to early unilateral nephrectomy, heart nuclear extracts showed increased histone H3 acetylation, H3K4me2, and H3S10ph, accompanied by upregulation of remodeling transcripts [112]. In this model, H3S10ph accompanies lysine acetylation and methylation during structural remodeling of the diabetic heart [112]. After MI, lactate accumulation activates TGF‐β/SMAD2 signaling and increases lactylation of snail family transcriptional repressor 1 (SNAI1), reinforcing a profibrotic endothelial‐to‐mesenchymal transition (EndMT) program and worsening cardiac fibrosis after MI [113]. In parallel, PAD4 is upregulated in infarcted myocardium and contributes to postinfarction fibrotic remodeling; in mouse MI, PAD4 deficiency reduces profibrotic gene expression and collagen deposition and improves ventricular function, supporting a maladaptive role for PAD4 in postinfarction fibrotic remodeling [114]. In pressure overload and aging models, PAD4‐dependent NETosis is linked to cardiac fibrotic remodeling [115]. Short‐chain enoyl‐CoA hydratase 1 (ECHS1) links mitochondrial metabolism to fibroblast chromatin remodeling. In this context, in ECHS1^+^/^−^ mice, primary cardiac fibroblasts show enhanced p300 nuclear translocation and p300‐dependent H3K9 acetylation, and ECHS1^+^/^−^ hearts develop diffuse interstitial fibrosis with increased α‐SMA, Collagen I, and Collagen III expression relative to wild‐type controls [116].

PTM‐linked pathways regulate the transcriptional programs that drive fibrogenic activation and ECM production in cardiac fibroblasts and relevant in vivo models. The strongest evidence involves acetylation‐ and methylation‐dependent control, particularly p300‐driven histone acetylation and H3K4, H3K9, and H3K27 methylation pathways. Lactylation, phosphorylation, and citrullination have also been implicated, supporting the view that fibrotic remodeling is governed through multiple chromatin layers rather than a single PTM axis. The key unresolved issue is which of these pathways are causal, cell‐type restricted, and tractable for antifibrotic intervention in vivo.

### 3.4 VSMC Phenotypic Switching

VSMCs retain substantial phenotypic plasticity in adult arteries. Under physiological conditions, medial VSMCs are quiescent and contractile, with high expression of myosin heavy chain 11 (MYH11) and actin alpha 2, smooth muscle (ACTA2) that support vasomotor tone. In response to hemodynamic stress, lipid accumulation, and inflammatory mediators, VSMCs downregulate contractile markers and adopt synthetic, inflammatory, osteogenic, or stem/progenitor‐like states [117, 118, 119]. These phenotypes contribute to neointimal hyperplasia, atherosclerotic plaque progression, and vascular calcification [117, 118, 120]. VSMC lineage‐tracing and single‐cell RNA sequencing (scRNA‐seq) studies show that many lesion‐associated cell states derive from the VSMC lineage [121, 122]. In AS and vascular calcification, altered histone methylation and acetylation remodel chromatin at contractile and osteogenic gene programs and shape VSMC identity during phenotypic switching [118, 120].

H3K4me2 at smooth muscle gene loci forms a lineage‐associated chromatin program that supports the VSMC contractile state and limits phenotypic drift [123]. In mouse arteries and cultured VSMCs, H3K4me2 is enriched at CArG box‐containing regulatory elements of myocardin‐dependent contractile genes [123]. The enrichment is retained during transient dedifferentiation and persists at poised loci despite changes in transcription, preserving lineage memory [123]. Myocd–LSD1 epigenome editing selectively reduces H3K4me2 at myocardin‐dependent contractile genes, including ACTA2, TAGLN, and MYH11 [123]. Redistribution of H3K4me2 toward stemness and developmental genes accompanies increased phenotypic plasticity in vitro and altered vascular remodeling in vivo [123]. H3K4me2 supports ten‐eleven translocation methylcytosine dioxygenase 2 (TET2) occupancy at the microRNA‐143/145 (miR‐143/145) locus, preserving 5‐hydroxymethylcytosine and miR‐145 expression during vascular remodeling [123].

Histone acetylation is a key determinant of whether VSMCs remain contractile or switch to synthetic states [120]. p300 and CBP are closely related HATs. They exert nonredundant and often opposing effects in human coronary artery VSMCs and in murine carotid ligation and restenosis models [120]. At CArG‐containing promoters of contractile genes, p300 cooperates with TET2. This maintains H3K9 and H3K27 acetylation, DNA hydroxymethylation, and RNA Pol II occupancy [120]. These changes sustain MYH11 and ACTA2 expression and limit VSMC proliferation, migration, and neointimal thickening [120]. CBP instead facilitates recruitment of HDAC2 and HDAC5 to contractile loci. This reduces histone acetylation and favors a synthetic, lesion‐promoting VSMC phenotype [120]. In human cardiac allograft vasculopathy, intimal hyperplasia lesions show reduced p300 and increased CBP expression compared with medial cells [120]. Vascular injury models also show that pELK1‐ and KLF4‐dependent recruitment of HDAC2 to the guanine/cytosine‐rich (G/C‐rich) repressor element in the SM22α promoter, together with recruitment of HDAC2 and HDAC5 to the smooth muscle α‐actin promoter, drives local histone deacetylation and repression of contractile genes during phenotypic switching [124, 125].

Repressive H3K9 methylation contributes to VSMC phenotypic switching. In murine carotid ligation, suppressor of variegation 3–9 homolog 1 (SUV39H1) expression and H3K9me3 increase in the media and neointima, whereas lysine demethylase 4A (KDM4A) is reduced. In human coronary artery VSMCs, SUV39H1 knockdown increases contractile gene expression and contractile function and reduces migration and proliferation. SUV39H1 is required for platelet‐derived growth factor‐induced KLF4 upregulation through miR‐143‐dependent control of KLF4 mRNA stability and regulation of chromatin at the KLF4 promoter, and it deposits H3K9me3 at contractile promoters to maintain repression. SUV39H1 loss increases KDM4A binding at contractile loci, reduces H3K9me3, and favors a contractile state [126].

Vascular injury and remodeling models indicate that chromatin regulation lies upstream of phenotypic switching during lesion formation. During disease progression, H3K4‐ and H3K9‐linked methylation programs, together with p300/CBP‐ and HDAC‐dependent acetylation, act on contractile, synthetic, and osteogenic gene modules, thereby biasing VSMCs toward either a stable contractile identity or lesion‐associated synthetic/osteogenic states that support lesion growth and calcification [119, 120, 123, 126].

### 3.5 Endothelial Dysfunction

Endothelial dysfunction is a core feature of AS, hypertension, and HF [127]. It is defined by reduced nitric oxide bioavailability, impaired barrier integrity, and increased endothelial expression of adhesion molecules, chemokines, and prothrombotic mediators [127]. ECs may undergo partial or complete EndMT, which contributes to neointima formation, perivascular fibrosis, plaque instability, and adverse cardiac remodeling [128, 129]. In vascular endothelium, histone PTMs integrate disturbed flow and chronic inflammation, producing chromatin remodeling at loci regulating nitric oxide production, barrier function, and mesenchymal programming [129, 130]. These PTM changes shift the endothelium from a homeostatic, NO‐producing phenotype toward persistent proinflammatory and profibrotic states in CVD [129, 130].

Histone H3 lysine methylation confers locus‐ and context‐specific control of endothelial transcription in CVD [131]. In EndMT models, IL‐1β together with TGF‐β2 downregulates EZH2 and reduces H3K27me3 at the TAGLN promoter, thereby inducing TAGLN; in ECs, EZH2‐dependent H3K27me3 represses endothelial adhesion and intercellular communication gene programs [132, 133]. Concurrently, EndMT stimuli induce the H3K9 demethylase JMJD2B (KDM4B), which removes H3K9me3 at promoters including calponin 1 (CNN1) and sulfatase 1 (SULF1) and drives an endothelial‐to‐mesenchymal transcriptional switch [134]. In coronary artery disease, coronary segments with greater intima–media thickness show reduced mitogen‐activated protein kinase 7 (MAPK7) and increased EZH2 expression [135]. In ECs, laminar shear activates MAPK7, increases miR‐101, and suppresses EZH2; this relieves EZH2‐dependent repression of the miR‐200 family and attenuates TGF‐β1‐induced EndMT, hyperpermeability, and collagen‐gel contraction [135]. Metabolic stress provides additional contexts in which histone methylation contributes to endothelial dysfunction. In obese mice, SUV39H1 is reduced and JMJD2C is increased in the vascular wall [136]. This is accompanied by reduced H3K9me2/3 and increased H3K9 acetylation at the p66Shc promoter [136]. p66Shc induction coincides with increased vascular ROS, nitric oxide depletion, and impaired endothelium‐dependent relaxation [136]. Restoring SUV39H1 re‐establishes H3K9 methylation at the p66Shc promoter, reduces p66Shc transcription, and improves endothelial function [136]. Transient hyperglycemia also leaves a persistent chromatin imprint. High glucose increases SET7/9‐dependent H3K4 monomethylation at the NF‐κB p65 promoter [137, 138]. NF‐κB‐dependent adhesion molecule and cytokine expression remains elevated after return to normoglycemia [137]. SET7/9‐driven H3K4 methylation at the NF‐κB p65 promoter provides a mechanistic basis for hyperglycemic “metabolic memory” and sustained proinflammatory endothelial programming in CVD [137, 138, 139].

Histone acetylation in ECs responds to hemodynamic and metabolic inputs. Shear stress, inflammatory mediators, and metabolic stress recalibrate the balance between acetyltransferases (including p300/CBP) and HDACs, remodeling chromatin accessibility at loci governing nitric oxide bioavailability, inflammatory activation, and endothelial–mesenchymal plasticity [128, 129, 130, 139]. In atheroprone regions, disturbed flow suppresses atheroprotective shear‐responsive programs and biases transcription toward NF‐κB‐dependent induction of adhesion molecules [127, 130]. Laminar shear promotes p300‐dependent histone H3/H4 acetylation at the endothelial nitric oxide synthase (eNOS) promoter and supports atheroprotective endothelial programs, including eNOS expression [127, 130]. The NAD^+^‐dependent deacetylase SIRT1 constrains oxidative stress and inflammatory gene expression through deacetylation of histone and nonhistone substrates [139]. In hyperglycemic and obesogenic contexts, reduced vascular SIRT1 expression or activity has been linked to mitochondrial dysfunction, endothelial activation, and low‐grade vascular inflammation [139]. In obesity‐related vascular disease, chronic metabolic overload and adipokine imbalance remodel histone acetylation at endothelial loci involved in oxidative‐stress responses and NO production, with increased H3K9 acetylation at the p66Shc promoter linked to ROS accumulation and NO depletion, consistent with the p66Shc‐centered chromatin shift described above [136]. Hemodynamic stress engages HDAC3 in the endothelium. In ApoE^−^/^−^ mice, HDAC3 is enriched in ECs at aortic branch points exposed to disturbed flow [61]. Endothelial Hdac3 silencing (shRNA) induces endothelial apoptosis and loss of endothelial integrity, with robust atherosclerotic lesion formation [61]. During EndMT, Class IIa HDAC9 is induced and represses endothelial identity genes while supporting mesenchymal programs [129]. Endothelial HDAC9 knockout reduces EndMT, plaque size, and lipid content and increases fibrous cap thickness, consistent with a more stable plaque phenotype [129]. Glycolysis‐driven lactate signals can be written into chromatin through H3K18 lactylation and reinforce EndMT programs [140].

In oxidized LDL‐stimulated human coronary artery ECs, glycolytic flux increases and intracellular lactate accumulates [140]. Lactate promotes p300/antisilencing function 1A histone chaperone (ASF1A)‐dependent H3K18 lactylation at the SNAI1 promoter, driving SNAI1 induction, EndMT marker expression, and barrier dysfunction [140]. Glycolysis inhibition or genetic targeting of lactate dehydrogenase A (LDHA), p300, or ASF1A reduces H3K18la, lowers SNAI1, and attenuates EndMT [140]. In ApoE^−^/^−^ mice, endothelial ASF1A deletion reduces endothelial H3K18la, limits EndMT, and decreases atherosclerotic lesion burden [140]. Extracellular histones, including NET‐associated species, directly perturb endothelial barrier function and inflammatory outputs [141, 142]. In human ECs, exogenous H3Cit disrupts barrier integrity by attenuating VE‐cadherin‐positive adherens junctions and inducing central actin stress fibers, accompanied by increased permeability [141]. Beyond barrier effects, native extracellular histones induce EC death and oxidative stress, suppress eNOS, and strongly upregulate IL‐6 and adhesion molecules in cultured human umbilical vein ECs [142]. In contrast, citrullinated histones preserve viability, do not induce oxidative stress, increase eNOS expression, and elicit a weaker inflammatory and adhesive program [142]. Disturbed flow drives proatherogenic endothelial transcription through the integrin α5β1–protein kinase N1 (PKN1)–histone H3.3 Ser31 phosphorylation (H3.3S31ph) axis [143]. PKN1 nuclear translocation and H3.3S31ph are required for FOS/FOSB induction and for full activation of adhesion and chemokine programs, including VCAM1, intercellular adhesion molecule 1 (ICAM1), E‐selectin (SELE), and C–C motif chemokine ligand 2 (CCL2) [143]. In vivo, endothelium‐specific PKN1 deletion or endothelial expression of H3.3S31A reduces disturbed‐flow‐induced endothelial inflammation and vascular remodeling [143].

Endothelial histone PTMs provide locus‐level routes by which hemodynamic and cardiometabolic stressors are written into sustained transcriptional states [130, 139, 143]. These PTMs converge on eNOS, NF‐κB, p66Shc, and EndMT control nodes, biasing NO bioavailability, barrier integrity, inflammatory activation, and mesenchymal plasticity in stressed vascular endothelium [129, 130, 136, 137, 140, 143]. Resolving how these chromatin and histone‐mediated inputs are integrated at key loci in vivo should clarify how endothelial dysfunction is sustained in vivo and help prioritize tractable chromatin regulators for intervention [130, 139].

### 3.6 Cardiac Hypertrophy and Remodeling

Pathological cardiac hypertrophy is a sustained stress response characterized by cardiomyocyte enlargement and myocardial remodeling that can progress to ventricular dilation and HF [144, 145]. The transition is accompanied by broad transcriptional reprogramming, including the reactivation of fetal and stress gene programs, such as natriuretic peptide A (NPPA; ANP), natriuretic peptide B (NPPB; BNP), and MYH7 [144, 146]. Metabolic remodeling contributes to progression toward maladaptive remodeling and HF [147, 148]. In cardiomyocytes, histone PTMs integrate Ca^2+^ signaling and metabolic state to shape chromatin regulation and transcription during remodeling [144, 145, 147].

Disruptor of telomeric silencing 1‐like (DOT1L) catalyzes H3K79me2, and cardiomyocyte H3K79me2 increases under hypertrophic stress [149]. Cardiomyocyte‐specific DOT1L loss blunts pressure overload‐induced remodeling, supporting a causal role for DOT1L in pathological hypertrophy [149]. DOT1L‐dependent H3K79me2 is enriched at the T‐box transcription factor 6 (TBX6) locus and increases TBX6 expression, promoting fetal gene activation and cardiomyocyte growth [149]. Other lysine methylation pathways implicated in hypertrophic signaling include PRC2‐associated EZH1/2‐dependent H3K27 methylation and multiple lysine demethylases [144]. Hypertrophic stimuli activate p300/CBP‐dependent acetylation, increase histone acetylation, and drive hypertrophy‐associated transcriptional programs [144, 150]. CBP or p300 overexpression elicits hypertrophic responses that require intact HAT activity [150]. In vivo, cardiac p300 promotes LV remodeling after MI in an acetyltransferase activity‐dependent manner [151]. Class IIa HDACs act as signal‐responsive repressors in cardiomyocytes, restraining prohypertrophic transcription and limiting myocyte enhancer factor 2 (MEF2)‐dependent gene activation [152]. Acetyl‐lysine reader proteins in the BET family, including bromodomain‐containing protein 4 (BRD4), interpret acetylated chromatin to support hypertrophy‐associated gene activation [8]. PRMT5 further augments p300‐dependent acetylation by methylating p300 and enhancing its HAT activity, with increased H3K9ac in PRMT5‐overexpressing hearts [153].

Ca2+‐dependent stress signaling also engages histone phosphorylation; calcium/calmodulin‐dependent protein kinase IIδ (CaMKIIδ) promotes histone H3 phosphorylation, including H3S10ph, to support hypertrophy‐associated gene expression programs [154]. Histone ubiquitination modulates hypertrophic control through H2A.Z regulation, with SIRT1 (Sir2α) promoting H2A.Z deacetylation followed by ubiquitin‐ and proteasome‐dependent turnover [155]. Tripartite motif‐containing protein 35 (TRIM35)‐mediated H2BK120ub1 promotes chromatin remodeling and P53‐dependent transcriptional activation in cardiomyocytes, with relevance to dilated cardiomyopathy (DCM) pathogenesis [156]. Metabolic remodeling is also reflected in lysine acylations that extend hypertrophic control beyond acetylation and methylation, with lactylation and crotonylation emerging as cardiomyocyte‐relevant marks [157]. In pressure overload models, Kla increases alongside pathological hypertrophy and has been connected to TGF‐β2 and phosphoinositide 3‐kinase (PI3K)–protein kinase B (AKT)–mechanistic target of rapamycin (mTOR) signaling [158]. Kcr has also been implicated in cardiomyocyte growth control; ECHS1 modulates crotonyl‐CoA availability and histone Kcr marks, including H3K18cr and H2BK12cr during hypertrophic remodeling [159].

Cardiac hypertrophy and remodeling involve chromatin programs that support reactivation of fetal and stress gene expression. Mechanistic evidence is strongest for acetylation and lysine methylation pathways, including p300/CBP HAT activity and DOT1L‐dependent H3K79me2 regulation of hypertrophic transcription [149, 150]. Additional layers include CaMKIIδ‐associated H3 phosphorylation, ubiquitin‐dependent regulation of H2A.Z and H2BK120ub1, and metabolite‐derived lactylation and crotonylation [154, 155, 156, 158, 159]. For several of these additional PTMs, locus‐resolved mechanisms and in vivo causal hierarchy remain incompletely defined [154, 157].

These mechanisms recur across major cardiovascular syndromes and provide context for interpreting how shared chromatin regulators contribute to HF, AS, MI, MIRI, and hypertension.

## 4 The Role of Histone Modification in CVD

CVDs arise from heterogeneous injury triggers and evolve through stage‐dependent cellular phenotypes. Building on the biochemical and mechanistic principles outlined in Section 3, this section examines histone PTMs across major cardiovascular conditions, with emphasis on evidence from in vivo perturbation studies and disease‐relevant phenotypes with translational implications. Table 1 summarizes the principal disease‐specific regulatory axes involving histone modifications across HF, AS, MI, MIRI, and hypertension.

**TABLE 1:** Regulatory mechanisms of histone posttranslational modifications across cardiovascular pathologies.

Histone modification	Modification function	Enzyme	Enzyme category	Histone target	Molecular axis	Impact on disease	References
Heart failure (HF)							
Methylation	Repressive	EZH2	Writer	H3K27me3	NEAT1–EZH2–Smad7 suppression	Promotes fibroblast activation and fibrosis	[160]
Methylation	Activating	NSD2	Writer	H3K36me2	NSD2–circCmss1–EIF4A3/TfR1–ferroptosis	Promotes hypertrophy, ferroptosis, and fibrosis	[161]
Methylation	Repressive	EHMT1/2	Writer	H3K9me2	miR‐217–EHMT1/2–fetal gene re‐expression	Restrains hypertrophy and fetal‐gene reactivation	[162]
Methylation	Repressive	SUV39H1	Writer	H3K9me2/3	Kindlin‐2–SUV39H1–GATA4 repression	Suppresses GATA4‐driven hypertrophy	[163]
Methylation	Repressive	SUV39H1	Writer	H3K9me3	MALAT1–SUV39H1–MyoD repression	Promotes hypertensive hypertrophy and fibrosis	[164]
Methylation	Activating	MLL3 (KMT2C)	Writer	H3K4me1	Carabin/STK24–CaN–MLL3–enhancer activation	Promotes hypertrophy and remodeling	[165]
Methylation	Activating	SETD2	Writer	H3K36me3	SETD2–SREBF1–lipotoxic injury	Promotes lipotoxic hypertrophy and diastolic dysfunction	[166]
Methylation	Activating	SETD7	Writer	H3K4me2/3	SETD7–E2F1–WWP2–GPX4 degradation	Promotes lipid peroxidation and hypertrophy	[167]
Demethylation	Context dependent	KDM1A (LSD1)	Eraser	H3K4me2, H3K9me2	LSD1–TGF‐β1–SMAD2/3	Context‐dependent; promotes fibrosis but preserves cardiomyocyte homeostasis	[168]
Demethylation	Activating	KDM3A	Eraser	H3K9me2	KDM3A–TIMP1	Promotes hypertrophy and fibrosis	[110]
Demethylation	Activating	KDM4A (JMJD2A)	Eraser	H3K9me3	JMJD2A–SRF/myocardin–FHL1	Promotes pathological hypertrophy	[169]
Demethylation	Repressive	KDM5 family	Eraser	H3K4me3	KDM5–ESRRA–FAO/OXPHOS	Impairs cardiomyocyte maturation; suppresses FAO/OXPHOS and myofibrillar organization	[170]
Deacetylation	Repressive	HDAC1/2/3	Eraser	/	Class I HDAC–TSC2 repression–mTOR activation	Promotes pathological hypertrophy and remodeling	[171]
Deacetylation	Repressive	HDAC1	Eraser	/	HDAC1–MEF2C–miR‐133a suppression	Promotes HHcy‐induced cardiomyocyte hypertrophy	[172]
Deacetylation	Repressive	HDAC2	Eraser	/	HDAC2–INPP5F repression–GSK3β inhibition	Promotes hypertrophy and fetal‐gene reactivation	[173]
Deacetylation	Repressive	HDAC3	Eraser	/	HDAC3–PINK1/Parkin‐mediated mitophagy	Promotes impaired mitophagy and cardiomyocyte apoptosis	[174]
Deacetylation	Repressive	HDAC8	Eraser	/	HDAC8–MMP12–NPPB	Promotes remodeling and fibrosis	[175]
Deacetylation	Repressive	HDAC8	Eraser	/	HDAC8–p38 MAPK	Promotes hypertrophy and fibrosis	[176]
Deacetylation	Repressive	SIRT3	Eraser	H3K27ac	SIRT3–FOS–AP‐1	Restrains inflammation and fibrosis	[177]
Deacetylation	Repressive	SIRT6	Eraser	H3K9ac	SIRT6–NF‐κB (p65)	Restrains Ang II‐induced cardiomyocyte hypertrophy	[178]
Acetylation	Activating	p300	Writer	H3K9ac	p300–H3K9ac–myofibroblast differentiation	Promotes hypertrophic and fibrotic remodeling.	[104]
Acetylation	Activating	p300	Writer	H3K9ac	p300–H3K9ac–collagen synthesis	Promotes fibroblast activation and fibrosis	[179]
Phosphorylation	Activating	MSK1/2	Writer (kinase)	H3S28ph	MSK1/2–BRG1/AP‐1 immediate‐early gene induction	Promotes immediate‐early gene induction and hypertrophy	[180]
Phosphorylation	Activating	SIRT1	Indirect regulator	γH2AX (Ser139)	SIRT1–H2AX deacetylation–DDR	Restrains DNA damage and cardiomyocyte death	[181]
Crotonylation	Activating	ECHS1	Indirect metabolic regulator	H3K18cr/H2BK12cr	ECHS1 deficiency–prohypertrophic gene induction	Promotes hypertrophy and remodeling	[159]
Atherosclerosis (AS)							
Methylation	Repressive	EZH2	Writer	H3K27me3	EZH2–SOCS3 repression–TLR/MyD88 signaling	Promotes TLR‐driven macrophage inflammation	[182]
Methylation	Repressive	EZH2	Writer	H3K27me3	GAS5–EZH2–ABCA1 repression	Promotes foam‐cell lipid accumulation and plaque progression	[183]
Methylation	Repressive	EZH2	Writer	H3K27me3	EZH2–IGFBP5 repression	Promotes endothelial inflammation and monocyte adhesion	[184]
Methylation	Repressive	EZH2	Writer	H3K27me3	EZH2–IL‐4/ZBTB16 repression	Promotes plaque progression by limiting Type 2 T‐cell plasticity	[185]
Demethylation	Activating	KDM6B (JMJD3)	Eraser	H3K27me3	KDM6B–profibrotic gene program	Promotes foam‐cell fibrotic phenotype	[186]
Methylation	Activating	SETD7	Writer	H3K4me1	SETD7–NF‐κB p65	Promotes endothelial inflammation and dysfunction	[138]
Methylation	Repressive	PRMT5	Writer	H3R8me2s, H4R3me2s	PRMT5–SMC marker repression	Promotes VSMC phenotypic switching and neointimal formation	[187]
Demethylation	Repressive	KDM1A	Eraser	H3K4me2	lnc_000048–KDM1A inhibition–MAP2K2/ERK activation	Promotes plaque inflammation and instability	[188]
Deacetylation	Repressive	HDAC3	Eraser	H3K9/14ac	HDAC3–TGF‐β1	Promotes plaque vulnerability	[62]
Deacetylation	Repressive	HDAC3	Eraser	H3K9ac	HDAC3–miR‐19b–PPAR‐γ/NF‐κB	Alleviates endothelial inflammation	[189]
Lactylation	Activating	p300	Writer	H3K18la	MCT4 deficiency–p300–reparative gene activation	Alleviates plaque inflammation	[190]
Lactylation	Activating	HDAC3	Eraser	H4K12la	TRAP1–HDAC3 inhibition–SASP activation	Promotes VSMC senescence	[191]
Lactylation	Activating	p300	Writer	H3K18la	ASF1A–p300–SNAI1 activation	Promotes EndMT	[140]
Citrullination	Activating	PAD4	Writer	H3Cit	PAD4–NETosis	Promotes arterial inflammation	[192]
Myocardial infarction (MI)							
Methylation	Repressive	EZH2	Writer	H3K27me3	MALAT1–EZH2–PPAR‐γ repression	Promotes macrophage inflammation and adverse remodeling	[193]
Methylation	Repressive	EZH2	Writer	H3K27me3	EZH2–DLL1/VEGF‐A/IRF4 repression	Promotes post‐MI inflammatory persistence and infarct expansion	[194]
Methylation	Repressive	SUV39H1	Writer	H3K9me3	SUV39H1–SIRT1 repression	Promotes ROS accumulation and cardiomyocyte death	[195]
Methylation	Repressive	SETD4	Writer	H4K20me3	SETD4–PI3K–Akt–mTOR–c‐Kit^+^ quiescence	Promotes cardiomyocyte apoptosis and impairs capillary repair	[196]
Methylation	Activating	DOT1L	Writer	H3K79me3	DOT1L–FoxO3a activation	Promotes fibroblast activation and postischemic fibrosis	[197]
Methylation	Activating	DOT1L	Writer	H3K79me2	DOT1L–SYK–TGF‐β1/Smad3 activation	Promotes fibroblast proliferation and fibrosis after MI	[198]
Demethylation	Repressive	KDM5B	Eraser	H3K4me2/3	KDM5B–ATF3–TGF‐β	Promotes post‐MI fibrosis and adverse remodeling	[199]
Lactylation	Activating	GCN5	Writer	H3K18la	IL‐1β–GCN5–LRG1/VEGF‐A/IL‐10	Promotes reparative monocyte programming and cardiac repair post‐MI	[76]
Citrullination	Activating	PAD4	Writer	H3Cit	PAD4–NETosis–S100A8/A9–TLR4/NLRP3–IL‐1β	Promotes neutrophil‐driven inflammation and granulopoiesis after MI	[200]
Myocardial ischemia–reperfusion injury (MIRI)							
Methylation	Repressive	EHMT2 (G9a)	Writer	H3K9me2	EHMT2–mTOR repression–autophagy	Alleviates MIRI through IPC‐dependent autophagy	[201]
Methylation	Repressive	SUV39H1	Writer	H3K9me3	SUV39H1–SIRT1 repression	Promotes oxidative injury and infarct damage in MIRI	[202]
Methylation	Activating	SETD7	Writer	H3K4 methylation	SETD7–Keap1–Nrf2/ARE	Promotes ROS and cardiomyocyte apoptosis in MIRI	[203]
Methylation	Activating	KMT2B	Writer	H3K4me3	KMT2B–RFK–TNF‐α/NOX2	Promotes ferroptosis, apoptosis, and inflammatory injury in MIRI	[204]
Deacetylation	Repressive	HDAC9	Eraser	/	HDAC9–Nrf2/Keap1/HO‐1	Promotes oxidative injury and cardiomyocyte apoptosis in MIRI	[205]
Demethylation	Activating	KDM3A	Eraser	H3K9me2	KDM3A–BRG1–NOX2/NOX4	Promotes endothelial ROS production in H/R	[206]
Lactylation	Activating	HSPA12A	Indirect regulator	H3K56la	HSPA12A–Smurf1–HIF‐1α	Alleviates cardiomyocyte death and dysfunction in MIRI	[95]
Ubiquitination	Repressive	PRC1	Writer	H2AK119ub	PRC1–HSP27–PFKFB3/COQ9	Promotes glycolytic impairment, mitochondrial ROS, and ferroptosis in MIRI	[207]
Hypertension							
Methylation	Repressive	EZH2	Writer	**/**	miR‐26a–EZH2–p21	Promotes VSMC proliferation and ECM deposition	[208]
Demethylation	Activating	KDM6B (JMJD3)	Eraser	H3K27me3	JMJD3–EDNRB/EDNRA–ERK	Restrains SMC phenotypic modulation and hypertensive arterial remodeling	[209]
Demethylation	Context dependent	KDM1A (LSD1)	Eraser	H3K4me1/2, H3K9me1/2	LSD1–MR/ENaC‐α/γ	Alleviates MR‐dependent salt‐sensitive hypertension	[210]
Methylation	Repressive	DOT1L	Writer	H3K79me2/3	DOT1L/AF9–ENaC‐α repression	Restrains ENaC‐α expression and renal sodium reabsorption	[211]

*Note*: This table summarizes the molecular axes linking specific histone writers, erasers, and other chromatin regulators to downstream pathological outcomes in heart failure (HF), atherosclerosis (AS), myocardial infarction (MI), myocardial ischemia–reperfusion injury (MIRI), and hypertension.

Abbreviations: ABCA1, ATP‐binding cassette transporter A1; AF9, ALL1‐fused gene from chromosome 9; Akt, protein kinase B; Ang II, angiotensin II; AP‐1, activator protein 1; ARE, antioxidant response element; ASF1A, antisilencing function 1A histone chaperone; ATF3, activating transcription factor 3; BNP, brain natriuretic peptide (NPPB); BRG1, brahma‐related gene 1; CaN, calcineurin; circCmss1, circular RNA derived from Cmss1; c‐Kit, KIT proto‐oncogene receptor tyrosine kinase; COQ9, coenzyme Q biosynthesis protein 9; DDR, DNA damage response; DLL1, delta‐like canonical Notch ligand 1; DOT1L, disruptor of telomeric silencing 1‐like; E2F1, E2F transcription factor 1; ECHS1, enoyl‐CoA hydratase, short chain 1; ECM, extracellular matrix; EDNRA/B, endothelin receptor type A/B; EHMT, euchromatic histone lysine methyltransferase; EIF4A3, eukaryotic translation initiation factor 4A3; ENaC, epithelial sodium channel; EndMT, endothelial‐to‐mesenchymal transition; ERK, extracellular signal‐regulated kinase; ESRRA, estrogen‐related receptor alpha; EZH2, enhancer of zeste homolog 2; FAO, fatty acid oxidation; FHL1, four and a half LIM domains 1; FOS, Fos proto‐oncogene, AP‐1 transcription factor subunit; G9a, euchromatic histone lysine methyltransferase 2; GATA4, GATA binding protein 4; GCN5, general control nonderepressible 5; GPX4, glutathione peroxidase 4; GSK3β, glycogen synthase kinase 3 beta; H/R, hypoxia/reoxygenation; H3Cit, citrullinated histone H3; H3K9ac, histone H3 lysine 9 acetylation; HDAC, histone deacetylase; HHcy, hyperhomocysteinemia; HIF‐1α, hypoxia‐inducible factor 1‐alpha; HO‐1, heme oxygenase 1; HSP27, heat shock protein 27; HSPA12A, heat shock protein family A member 12A; IGFBP5, insulin‐like growth factor binding protein 5; IL, interleukin; IL‐10, interleukin 10; IL‐1β, interleukin 1 beta; IL‐4, interleukin 4; INPP5F, inositol polyphosphate‐5‐phosphatase F; IPC, ischemic preconditioning; IRF4, interferon regulatory factor 4; JMJD, Jumonji domain‐containing protein; KDM, lysine demethylase; Keap1, kelch‐like ECH‐associated protein 1; KMT, lysine methyltransferase; lnc_000048, long noncoding RNA 000048; LRG1, leucine‐rich alpha‐2‐glycoprotein 1; LSD1, lysine‐specific demethylase 1; MAP2K2, mitogen‐activated protein kinase kinase 2; MAPK, mitogen‐activated protein kinase; MCT, monocarboxylate transporter; MCT4, monocarboxylate transporter 4; MEF2C, myocyte enhancer factor 2C; MI, myocardial infarction; MIRI, myocardial ischemia–reperfusion injury; MMP, matrix metalloproteinase; MR, mineralocorticoid receptor; MSK1/2, mitogen‐ and stress‐activated protein kinase 1/2; mTOR, mechanistic target of rapamycin; MyD88, myeloid differentiation primary response 88; MyoD, myoblast determination protein 1; NEAT1, nuclear paraspeckle assembly transcript 1; NETosis, neutrophil extracellular trap formation; NF‐κB, nuclear factor kappa B; NLRP3, NLR family pyrin domain containing 3; NOX, NADPH oxidase; Nrf2, nuclear factor erythroid 2‐related factor 2; NSD2, nuclear receptor binding SET domain protein 2; other histone marks are denoted using the same residue‐specific nomenclature (eg, H3K27me3, H3K36me2, H3K18la, H2AK119ub); OXPHOS, oxidative phosphorylation; p21, cyclin‐dependent kinase inhibitor 1A; p65, RELA/p65 subunit of NF‐κB; PAD4, peptidylarginine deiminase 4; Parkin, parkin RBR E3 ubiquitin protein ligase; PFKFB3, 6‐phosphofructo‐2‐kinase/fructose‐2,6‐bisphosphatase 3; PI3K, phosphoinositide 3‐kinase; PINK1, PTEN‐induced kinase 1; PPAR‐γ, peroxisome proliferator‐activated receptor gamma; PRC, polycomb repressive complex; PRC1, polycomb repressive complex 1; PRMT5, protein arginine methyltransferase 5; RFK, riboflavin kinase; ROS, reactive oxygen species; S100A8/A9, S100 calcium‐binding proteins A8/A9; SASP, senescence‐associated secretory phenotype; SETD, SET domain containing; SIRT, sirtuin; SMAD, suppressor of mothers against decapentaplegic; SMC, smooth muscle cell; Smurf1, SMAD specific E3 ubiquitin protein ligase 1; SNAI1, snail family transcriptional repressor 1; SOCS3, suppressor of cytokine signaling 3; SREBF1, sterol regulatory element binding transcription factor 1; SRF, serum response factor; STK24, serine/threonine kinase 24; SUV39H1, suppressor of variegation 3–9 homolog 1; SYK, spleen tyrosine kinase; TfR1, transferrin receptor 1; TGF‐β, transforming growth factor beta; TIMP1, tissue inhibitor of metalloproteinases 1; TLR, Toll‐like receptor; TLR4, Toll‐like receptor 4; TNF‐α, tumor necrosis factor alpha; TRAP1, TNF receptor‐associated protein 1; TSC2, tuberous sclerosis complex 2; VEGF‐A, vascular endothelial growth factor A; VHL, von Hippel–Lindau; VSMC, vascular smooth muscle cell; WWP2, WW domain containing E3 ubiquitin protein ligase 2; ZBTB16, zinc finger and BTB domain containing 16; γH2AX, phosphorylated H2AX at serine 139.

### 4.1 Heart Failure

**FIGURE 3:**
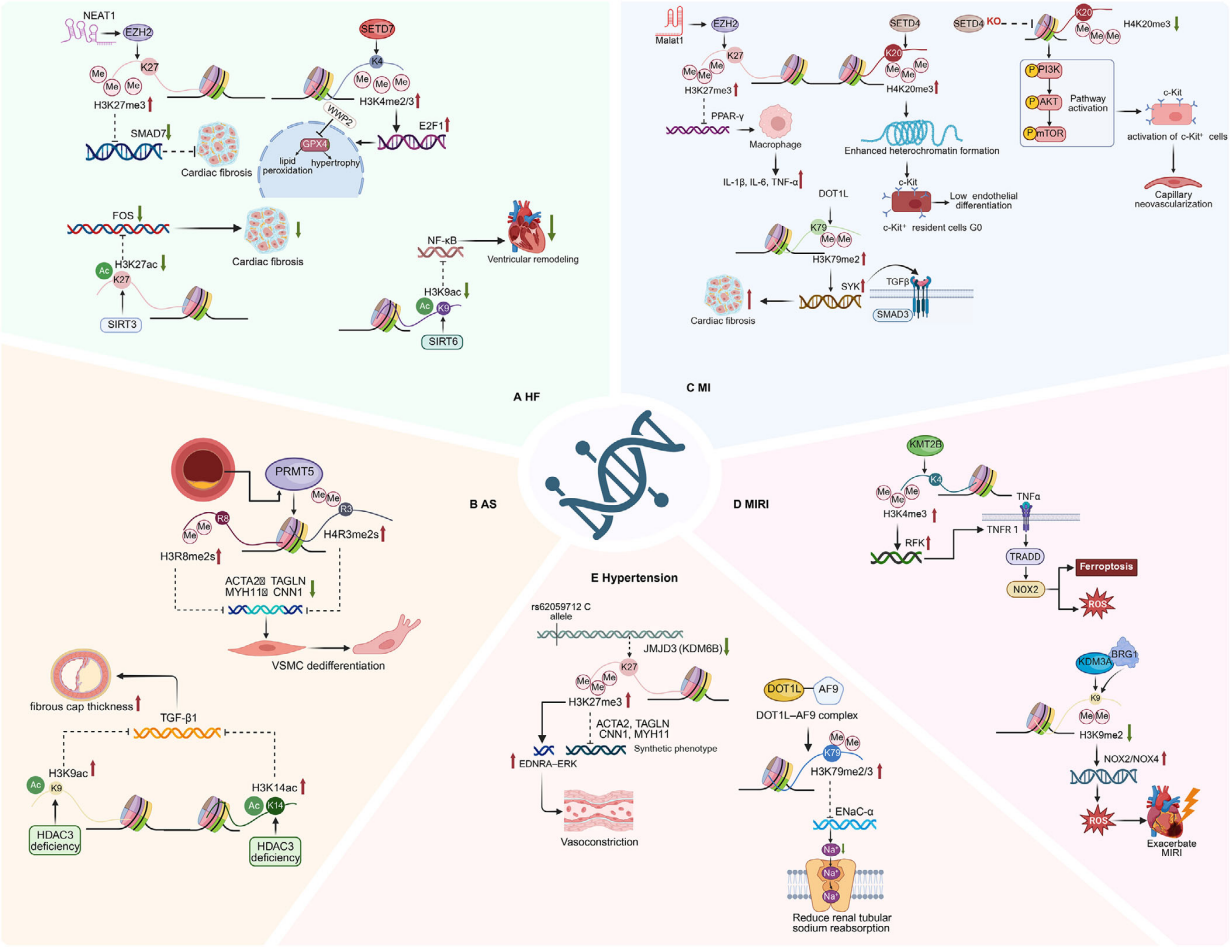
Representative histone modification axes across major cardiovascular pathologies. Schematic summary of selected epigenetic regulators and histone modification axes associated with phenotypic change in (A) heart failure (HF), (B) atherosclerosis (AS), (C) myocardial infarction (MI), (D) myocardial ischemia–reperfusion injury (MIRI), and (E) hypertension. The figure highlights representative upstream regulators, principal downstream targets, and major pathological or protective consequences in each disease context. AF9, ALL1‐fused gene from chromosome 9; AKT, protein kinase B; AS, atherosclerosis; BRG1, Brahma‐related gene 1; DOT1L, disruptor of telomeric silencing 1‐like; ECM, extracellular matrix; E2F1, E2F transcription factor 1; EDNRA, endothelin receptor type A; ENaC‐α, epithelial sodium channel alpha subunit; ERK, extracellular signal‐regulated kinase; EZH2, enhancer of zeste homolog 2; GPX4, glutathione peroxidase 4; HDAC, histone deacetylase; HF, heart failure; JMJD3, Jumonji domain‐containing protein 3; KDM3A, lysine demethylase 3A; KMT2B, lysine methyltransferase 2B; Malat1, metastasis‐associated lung adenocarcinoma transcript 1; MI, myocardial infarction; MIRI, myocardial ischemia–reperfusion injury; NOX, NADPH oxidase; PI3K, phosphoinositide 3‐kinase; PRMT5, protein arginine methyltransferase 5; RFK, riboflavin kinase; ROS, reactive oxygen species; SETD4, SET domain‐containing lysine methyltransferase 4; SETD7, SET domain‐containing lysine methyltransferase 7; SIRT3, sirtuin 3; SIRT6, sirtuin 6; SMAD3, SMAD family member 3; SYK, spleen tyrosine kinase; TNF‐α, tumor necrosis factor alpha; TNFR1, tumor necrosis factor receptor 1; TRADD, TNFRSF1A‐associated via death domain; TGF‐β, transforming growth factor beta; VSMC, vascular smooth muscle cell; WWP2, WW domain‐containing E3 ubiquitin protein ligase 2. This figure was created by the authors with BioRender.com and does not reproduce any copyrighted third‐party published material.

HF is a clinical syndrome caused by structural or functional cardiac abnormalities that impair ventricular filling or ejection [212]. It often develops as the consequence of sustained maladaptive ventricular remodeling [213]. As discussed in Section 3.6, chromatin‐based epigenetic regulation contributes to cardiomyocyte transcriptional reprogramming, including reactivation of fetal and stress‐responsive gene programs [152, 213]. In parallel, fibroblast activation with ECM deposition drives interstitial and perivascular fibrosis in HF [213, 214] (Figure 3A).

#### 4.1.1 Histone Methylation Modification and HF

Methylation dynamics orchestrate the transition from adaptive compensation to decompensated HF by regulating distinct pathological modules. EZH2‐mediated H3K27me3 serves as a central driver of myofibroblast activation; in failing hearts, the lncRNA nuclear paraspeckle assembly transcript 1 (NEAT1) recruits EZH2 to the SMAD7 promoter, amplifying TGF‐β signaling and exacerbating fibrosis, a phenotype reversed by NEAT1 silencing [160]. Converging on the same fibroproliferative program, the demethylase JMJD3 (KDM6B) removes repressive H3K27me3 at the β‐catenin promoter, activating Wnt signaling to further promote fibroblast activation [215]. The demethylase KDM1A (LSD1) likewise exhibits a cell‐type‐specific dichotomy in vivo. Myofibroblast‐specific LSD1 deletion attenuates TGF‐β1–SMAD2/3 signaling, fibrosis, and systolic dysfunction, whereas cardiomyocyte‐specific LSD1 loss induces mild hypertrophy and dysfunction, underscoring the complexity of targeting this enzyme [168].

Distinct from these fibrotic mechanisms, pathological hypertrophy involves a shift in the balance of H3K9, H3K4, and H3K36 methylation. At homeostasis, euchromatic histone KMT 1/2 (EHMT1/2) maintains the repression of fetal genes (NPPA, MYH7) via H3K9me2, but stress‐induced miR‐217 disrupts this protective mechanism [162]. Conversely, NSD2 promotes pathological hypertrophy [161]. Its prohypertrophic effects have been linked to circCmss1/EIF4A3/TfR1‐associated ferroptotic signaling [161]. The H3K4 monomethyltransferase MLL3 (KMT2C) also promotes pressure overload‐induced hypertrophy and remodeling by coupling calcineurin signaling to enhancer activation; cardiac MLL3 knockdown blunts this maladaptive program [165]. H3K9 methylation pathways are likewise context dependent; for instance, SUV39H1 blunts hypertrophy when recruited by Kindlin‐2 [163] but drives maladaptive remodeling in hypertensive models via MALAT1 (metastasis‐associated lung adenocarcinoma transcript 1)‐dependent recruitment of SUV39H1 to MyoD‐binding loci [164]. Among demethylases, KDM4A promotes hypertrophy by cooperating with serum response factor/myocardin to induce FHL1 (four‐and‐a‐half LIM domains 1) expression [169], while KDM3A promotes pathological remodeling by activating cardiomyocyte TIMP1 transcription, supporting a paracrine profibrotic role [110].

Methylation defects are increasingly implicated in myocardial metabolic remodeling, particularly in cardiometabolic HF with preserved ejection fraction (HFpEF). The H3K36me3 writer SETD2 is upregulated in HFpEF models, targeting the SREBF1 promoter to drive lipotoxicity and diastolic dysfunction [166]. Similarly, SETD7‐mediated H3K4 methylation activates E2F1, which in turn upregulates the ubiquitin ligase WWP2; WWP2 then degrades the antioxidant glutathione peroxidase 4 (GPX4), leading to lipid peroxidation and hypertrophy [167]. In contrast, the methyltransferase SMYD1 is downregulated at the RNA level in human DCM, correlating with reduced H3K4me3 at SMYD1‐enriched promoters, including PPARGC1A [216]. KDM5 inhibition restores cardiomyocyte maturation and oxidative phosphorylation [199]. Loss of KDM5B also derepresses activating transcription factor 3 (ATF3) and limits fibrosis [170].

#### 4.1.2 Histone Acetylation Modification and HF

Acetylation dynamics coordinate the transition to HF by regulating hypertrophic, fibrotic, and electrical remodeling programs. Class I HDAC activity generally favors maladaptive remodeling. Mechanistically, Class I HDAC activity (notably HDAC1/2/3) suppresses the antihypertrophic mTOR inhibitor tuberous sclerosis complex 2 (TSC2) [171]. In HHcy cardiomyocytes, HDAC1 forms repressive complexes with MEF2C to suppress miR‐133a, and H_2_S disrupts this interaction to restore miR‐133a expression [172]. Within this class, HDAC8 specifically promotes pathological remodeling by activating the p38 MAPK pathway [176] and is associated with increased MMP12 and NPPB expression [175]. However, HDAC2 exhibits a stage‐dependent duality. Its deletion confers resistance to hypertrophic stimulation by preserving glycogen synthase kinase 3β (GSK3β) activity [173], whereas its downregulation is linked to ventricular electrical remodeling in early HF [217]. p300‐dependent acetylation also contributes to fibrotic remodeling. Pharmacological inhibition of p300 (e.g., C646, L002) lowers myocardial histone acetylation, suppresses myofibroblast differentiation, and attenuates Ang II‐ or hypertension‐associated hypertrophic and fibrotic remodeling without reducing elevated blood pressure [104, 179].

Distinct from these maladaptive drivers, Class IIa HDACs (HDAC4, HDAC5, and HDAC9) act as signal‐responsive “brakes” on hypertrophy. Under stress, their nuclear export relieves the repression of MEF2, reactivating fetal gene programs. This export event disrupts an HDAC4‐associated SUV39H1/HP1 repressive state, relieving H3K9 methylation‐dependent silencing at the NPPA and NPPB promoters [218].

Beyond structural remodeling, deacetylation also influences mitochondrial quality control. In hypoxic H9c2 cardiomyocytes, selective inhibition of HDAC3 (via RGFP966) restores PINK1/Parkin‐mediated mitophagy and reduces apoptosis [174]. SIRTs also contribute protective counter‐regulation; SIRT3 deacetylates H3K27ac at the Fos promoter to limit fibrosis [177], while SIRT6 deacetylates H3K9 and suppresses NF‐κB‐dependent transcription, thereby limiting maladaptive stress signaling [178, 219].

#### 4.1.3 Histone Phosphorylation and Metabolite‐Derived Acylations in HF

Beyond classical marks, phosphorylation and metabolite‐derived acylations can mark distinct stress states. H3S28 phosphorylation increases in end‐stage human HF [220], and in experimental models it couples stress signaling to immediate‐early gene induction during hypertrophic remodeling [180]. In models of DOX cardiotoxicity, SIRT1 counteracts myocardial injury by regulating H2AX phosphorylation and attenuating oxidative stress [181]. Histone lactylation has been detected in hypertrophic and failing myocardium, whereas Kcr has been reported in hypertrophic myocardium [158, 159], linking metabolic remodeling in the stressed heart to chromatin plasticity.

In HF, the clearest histone mechanisms still center on methylation‐ and acetylation‐dependent control of fetal gene reactivation, fibroblast activation, and maladaptive remodeling. By contrast, phosphorylation, lactylation, and crotonylation appear to track more dynamic stress and metabolic states in the failing heart, but for most of these marks, it remains unresolved whether they act as upstream drivers of disease progression or as downstream signatures of sustained remodeling.

### 4.2 Atherosclerosis

AS represents a continuum of maladaptive inflammatory and structural remodeling driven by dyslipidemia [221, 222]. The progression from stable fatty streaks to vulnerable plaques involves dynamic chromatin remodeling that shapes endothelial activation, macrophage polarization, and VSMC plasticity [53, 222]. Although lipid retention and local vascular stress initiate the disease, epigenetic mechanisms, particularly histone methylation and acetylation, shape the transcriptional programs that underlie plaque progression and dictate the critical balance between lesion stability and vulnerability [53] (Figure 3B).

#### 4.2.1 Histone Methylation Modification and AS

Methylation marks integrate metabolic stress, hemodynamic cues, and vascular inflammation. The methyltransferase EZH2 contributes to plaque progression through cell‐type‐specific H3K27me3 repression programs. In macrophages, EZH2‐mediated H3K27me3 can repress SOCS3 and sustain TLR‐induced MyD88‐dependent NF‐κB signaling [182], and myeloid EZH2 deficiency reduces atherosclerotic lesion size and dampens foam cell inflammatory responses in vivo [223]. The lncRNA GAS5 recruits EZH2 to deposit H3K27me3 at the ATP‐binding cassette transporter A1 (ABCA1) promoter, repressing ABCA1 transcription and cholesterol efflux and thereby favoring foam cell formation [183]. In endothelium, atherosusceptible regions exposed to disturbed flow display higher EZH2 expression than laminar flow regions, and flow‐dependent EZH2 and H3K27me3 regulation contribute to repression of anti‐inflammatory targets such as insulin‐like growth factor–binding protein 5 (IGFBP5), supporting lesion‐prone endothelial phenotypes [184]. In the adaptive immune compartment, CD4^+^ T cell‐specific EZH2 deficiency promotes Type 2 immune responses with increased IL‐4 and is accompanied by reduced plaque burden [185]. Conversely, the H3K27me3 demethylase KDM6B (JMJD3) supports a profibrotic transcriptional program in macrophage foam cells, with KDM6B deficiency suppressing profibrotic and ECM/collagen programs in macrophage foam cells [186].

Distinct from these H3K27me3 dynamics, arginine methylation critically regulates VSMC plasticity. PRMT5 is upregulated in human atherosclerotic lesions and deposits symmetric dimethylarginine marks (H3R8me2s/H4R3me2s) that silence contractile genes (ACTA2, MYH11), driving phenotypic switching and neointimal hyperplasia [187]. Balancing these writer activities, demethylases also modulate disease outcomes; in ApoE^−/−^ mice, pharmacological inhibition of KDM1A (LSD1) reduces atherosclerotic lesion formation with decreased oxidative stress and inflammatory markers [74]. In carotid AS, lnc_000048 interacts with KDM1A to enhance MAP2K2–ERK signaling and inflammatory and matrix‐degrading outputs, thereby reducing plaque stability [188].

#### 4.2.2 Histone Acetylation Modification and AS

Acetylation dynamics critically influence fibrous cap composition and lesion stability, with HDAC3 functioning as a pivotal, cell‐type‐specific regulator. In macrophages, HDAC3 acts as a brake on alternative activation. Although myeloid HDAC3 deletion promotes a stable plaque phenotype (thicker fibrous caps) via increased TGF‐β1 secretion, it paradoxically increases overall lesion size [62]. Clinically, HDAC3 is upregulated in human ruptured plaques and correlates inversely with TGF‐β1, consistent with plaque vulnerability [62].

Distinct from its role in macrophages, endothelial HDAC3 exerts protective anti‐inflammatory effects by suppressing miR‐19b through reduced H3K9 acetylation at its promoter, thereby preserving PPAR‐γ expression and attenuating endothelial inflammatory signaling [189].

#### 4.2.3 Other Histone Modifications and AS

Metabolic and immune‐derived marks provide novel regulatory layers to atherosclerotic progression. Kla operates in a highly cell‐type‐specific manner. In macrophages, lactate‐driven H3K18la supports a reparative, anti‐inflammatory phenotype that limits plaque progression [190]. This contrasts sharply with VSMCs, where H4K12la is linked to senescence and accelerated lesion growth [191], underscoring the context‐dependence emphasized in Section 3.6. H3Cit marks NETs within plaques. In ApoE^−/−^ mice, PAD inhibition with Cl‐amidine blocks NET formation, reduces atherosclerotic lesion area, and delays carotid artery thrombosis [55], whereas myeloid‐specific PAD4 deletion diminishes NET formation and reduces atherosclerotic burden [192].

In AS, histone dysregulation does not operate through a single uniform transcriptional program, but through progressive reprogramming of macrophages, endothelium, and VSMCs that determines whether plaques enlarge, stabilize, or become rupture‐prone.

### 4.3 Myocardial Infarction

MI initiates profound metabolic and inflammatory shifts [224]. In this ischemic context, chromatin‐based regulators contribute to early inflammatory activation and subsequent remodeling responses, including fibrotic repair [224] (Figure 3C).

#### 4.3.1 Histone Methylation Modification and MI

Histone methylation writers and erasers participate in distinct phases of post‐MI repair, spanning inflammatory regulation, fibrotic remodeling, and endogenous reparative neovascularization [193, 194, 195, 196, 197, 198, 199].

During the initial inflammatory phase, epigenetic writers drive immune cell activation. The lncRNA Malat1 binds EZH2 and increases H3K27me3, thereby suppressing PPAR‐γ expression and aggravating macrophage‐associated inflammation after AMI [193]. Consistently, pharmacological EZH2 inhibition with GSK‐343 accelerates inflammatory resolution, reduces infarct scar expansion, and improves cardiac function post‐MI [194].

As the infarct transitions to the remodeling phase, methyltransferases facilitate fibroblast activation and scar formation. DOT1L‐mediated H3K79me3 at the FoxO3a promoter activates FoxO3a transcription and downstream profibrotic signaling in cardiac fibroblasts [197], and DOT1L‐mediated H3K79me2 at the spleen tyrosine kinase (SYK) promoter activates TGF‐β1/Smad3 signaling to promote fibroblast proliferation and fibrosis after MI [198]. Working alongside these writers, KDM5B demethylates H3K4me2/3 at the ATF3 promoter, represses ATF3, and enhances TGF‐β signaling; KDM5B deficiency reduces scar size and improves ejection fraction after MI [199].

Beyond fibrotic remodeling, histone methylation also shapes cardiomyocyte survival and endogenous reparative neovascularization after MI. SUV39H represses the cardioprotective gene SIRT1 through H3K9me3 at the SIRT1 promoter, thereby worsening ischemic injury [195]. Distinct from survival pathways, SETD4 acts as an epigenetic brake on endogenous c‐Kit+ cell activation by maintaining quiescence through H4K20me3. Its deletion promotes capillary neovascularization, limits infarct size, and improves cardiac function after MI [196].

#### 4.3.2 Other Histone Modifications and MI

Other PTMs also shape the balance between inflammatory amplification and reparative immune responses after MI. Glycolysis‐derived lactate induces Kla (H3K18la) in post‐MI monocytes and macrophages, driving reparative genes such as Lrg1 and Vegf‐a that promote angiogenesis and functional recovery [76]. By contrast, PAD4‐dependent H3Cit marks NETosis and is linked to early S100A8/A9 release that amplifies acute post‐MI inflammation [200]. In STEMI patients, circulating H3Cit–DNA complexes are independently associated with worse outcomes, particularly higher MACE risk, after STEMI [81].

Mechanistic evidence for histone Kla‐linked reparative programs and PAD4–H3Cit–NETosis after MI still comes largely from experimental immune‐cell and in vivo studies. Current human evidence is mainly biomarker‐based, which supports clinical relevance but does not yet establish causality.

Throughout the postinfarct course, histone PTMs are linked to the transition from inflammatory injury to fibrotic repair and reparative neovascularization. The mechanistic basis is clearest for methylation pathways, whereas evidence for nonclassical marks remains more limited and less clearly causal [81, 193, 194, 195, 196, 198, 199, 200].

### 4.4 Myocardial Ischemia–Reperfusion Injury

Reperfusion paradoxically exacerbates injury through excessive ROS generation and calcium overload [202]. Within this acute stress window, rapid chromatin remodeling governs the critical balance between cardiomyocyte survival mechanisms, such as autophagy [201], and lethal pathways including ferroptosis and mitochondrial injury [202, 207] (Figure 3D).

#### 4.4.1 Histone Methylation and Deacetylation in MIRI

Methylation dynamics differentially regulate cardiomyocyte survival pathways, oxidative thresholds, and sex‐linked determinants of ischemic tolerance. Ischemic preconditioning engages a protective G9a‐mediated H3K9me2 program that increases H3K9me2 throughout the Mtor gene, represses Mtor, and induces autophagy [201]. Conversely, endothelial H3K9me3 rises during mid‐reperfusion; silencing SUV39H1 or overexpressing KDM4D reduces H3K9me3, partially restores endothelial function‐related gene expression, and rescues endothelial tube formation [225]. In cardiomyocytes, SUV39H1 is enriched at the SIRT1 promoter and increases H3K9me3, whereas SUV39H1 deficiency or inhibition restores SIRT1 and attenuates oxidative stress and myocardial I/R injury [202].

Beyond these survival switches, methylation regulators critically tune the oxidative threshold during reperfusion. SETD7 exacerbates injury by dampening Keap1/Nrf2/ARE antioxidant signaling; silencing SETD7 decreases Keap1, activates Nrf2/ARE readouts, and reduces ROS and apoptosis [203], while KMT2B promotes ferroptosis and apoptosis through H3K4me3‐dependent RFK transcription and downstream activation of the TNF‐α/NOX2 axis [204]. Histone deacetylation also modulates redox defense in I/R‐related myocardial injury. HDAC9 acts as a maladaptive brake on the Nrf2–Keap1–HO‐1 antioxidant axis, and its knockdown attenuates cardiomyocyte apoptosis and myocardial I/R injury [205]. In parallel, the KDM3A–BRG1 complex modulates endothelial ROS generation by regulating NOX2/NOX4 transcription in ECs [206].

Sex‐specific disparities in ischemic tolerance may also reflect chromatin‐linked effects of sex chromosome dosage. KDM6A, one of several X chromosome escape genes expressed at higher levels in XX than XY hearts, was proposed as a candidate contributor to sex chromosome dose effects on mitochondrial calcium retention capacity and mPTP threshold, without direct causal proof [226].

#### 4.4.2 Other Histone Modifications and MIRI

Metabolic and repressive histone marks critically influence reperfusion outcomes. During reoxygenation, maintenance of aerobic glycolytic flux sustains H3K56la in cardiomyocytes; this mark is required for the HSPA12A‐mediated prosurvival response after hypoxia–reoxygenation, because blocking lactate generation with oxamate or p300 activity with C646 abolishes both H3K56la maintenance and the protective effect [95].

Histone ubiquitination also regulates lethal signaling. The PRC1 increases H2AK119ub occupancy at the Hsp27 locus, repressing its expression. Pharmacological inhibition of PRC1 restores Hsp27, which stabilizes COQ9, limits ROS generation, and suppresses ferroptotic cell death [207].

In MIRI, histone PTMs regulate several processes that bear directly on tissue outcome, including autophagy, endothelial recovery, redox balance, susceptibility to ferroptotic injury, and sex‐linked variation in ischemic tolerance. Although the supporting evidence remains predominantly preclinical, the recurrence of these mechanisms across distinct histone marks indicates that chromatin regulation participates directly in reperfusion injury rather than simply mirroring cellular stress [201, 202, 203, 204, 205, 206, 207, 225, 226].

### 4.5 Hypertension

Systemic hypertension is sustained by the interplay between maladaptive vascular remodeling and disordered renal sodium handling [210, 211, 227]. Epigenetic mechanisms contribute to the transcriptional reprogramming that underlies these phenotypes, including endothelial dysfunction, VSMC plasticity, inflammatory signaling, and epigenetically regulated sodium‐handling pathways [210, 211, 228] (Figure 3E).

#### 4.5.1 Histone Methylation Modification and Hypertension

Epigenetic writers and erasers modulate blood pressure homeostasis through two principal mechanisms, one governing vascular resistance and the other controlling renal sodium handling.

In the vessel wall, EZH2 drives pathological remodeling. Downregulation of miR‐26a derepresses EZH2, which promotes VSMC proliferation and ECM deposition; restoring miR‐26a restrains this axis and attenuates hypertensive vascular remodeling [208]. Beyond this proliferative axis, JMJD3 (KDM6B) restrains smooth muscle phenotypic modulation and hypertensive arterial remodeling. In a hypertension‐associated genome‐wide association study, the major T allele of rs62059712 reduces JMJD3 expression, shifts endothelin signaling toward the vasoconstrictive EDNRA subtype and away from EDNRB, suppresses contractile gene programs, and enhances ERK activation; pharmacological blockade of EDNRA normalizes the hypertensive and contractile consequences of JMJD3 loss [209].

Distinct from these vascular mechanisms, methylation in the kidney dictates volume status. DOT1L, through the Dot1a–Af9 repressor complex, limits ENaC‐α transcription by promoting H3K79 hypermethylation at the ENaC‐α promoter. Aldosterone‐induced Sgk1 disrupts the Dot1a–Af9 interaction, leading to targeted H3K79 hypomethylation and derepression of ENaC‐α transcription [211]. Similarly, KDM1A (LSD1) limits mineralocorticoid receptor (MR)‐dependent sodium retention; its deficiency elevates renal MR and α‐/γ‐ENaC expression, producing salt‐sensitive hypertension that is responsive to eplerenone [210].

#### 4.5.2 Histone Acetylation Modification and Hypertension

Increased HDAC activity acts as a central node linking inflammation to hypertensive remodeling. In the SHR, generalized HDAC hyperactivation sustains an inflammatory feedback loop involving NF‐κB signaling and NOX2‐derived ROS. Pharmacological inhibition with valproic acid effectively lowers blood pressure and attenuates ventricular hypertrophy. Mechanistically, this protection is driven by the reduced cardiac Ang II Type 1 Receptor expression and the suppression of oxidative stress, establishing a direct link between chromatin state and the renin–Ang system [228].

However, the chromatin landscape exhibits significant heterogeneity across hypertensive etiologies. Specific isoforms show divergent patterns across hypertensive models. For instance, HDAC3 is upregulated in genetic models such as the SHR, whereas HDAC8 is reduced in both the SHR and Ang II‐induced hypertension models [229]. This underscores the stimulus‐dependent nature of epigenetic reprogramming in vascular pathology.

#### 4.5.3 Other Histone Modifications and Hypertension

Kla links lactate‐dependent metabolic reprogramming to vascular remodeling. In pulmonary hypertension, hypoxia‐driven glycolytic reprogramming increases lactate availability and enhances H3K18la at HIF‐1α target loci linked to proliferative remodeling programs in pulmonary artery smooth muscle cells. In parallel, H3K18la and H4K5la are increased in hypoxic pulmonary arteries and lung tissues, and inhibition of lactate production with the LDH inhibitor oxamate reduces these marks and attenuates hypoxia‐driven pulmonary vascular remodeling [99]. While currently defined primarily in the pulmonary circulation, this metabolic–epigenetic axis remains to be tested in systemic arterial hypertension. In systemic hypertension, histone PTMs converge most clearly on vascular remodeling, inflammatory signaling, and renal sodium handling, with functional effects that remain strongly context dependent.

Across CVDs, histone PTMs converge on a limited set of pathological processes, including cardiomyocyte stress adaptation, fibroblast activation, endothelial dysfunction, vascular smooth muscle phenotypic switching, inflammatory programming in immune cells, and renal sodium handling. Their effects remain strongly context dependent and vary with cell type, disease stage, and lesion phenotype.

## 5 Therapeutic Targeting of Histone Modifications in CVD

Several chromatin‐modifying enzymes and reader proteins have tractable chemical probes and, in some cases, clinical‐grade inhibitors. This creates an opportunity to test whether perturbing defined histone PTM pathways can reshape cardiovascular remodeling. Interpretation depends on isoform selectivity, off‐target activity, and pharmacodynamic (PD) evidence that the intended histone mark is engaged in the relevant tissue and cellular compartment. We therefore summarize the main therapeutic entry points and the evidence supporting each approach. Table 2 summarizes representative chromatin‐targeting strategies and their current translational status in CVD.

**TABLE 2:** Translational landscape and development status of selected chromatin‐targeting strategies in cardiovascular disease.

Target class	Targeted modification	Representative agents/interventions	Cardiovascular application prospects/theoretical basis	CVD clinical development status	Overall clinical development status	Dose‐limiting or notable toxicities	References
HDAC inhibition	Histone deacetylation and acetylation‐dependent transcription	Valproic acid; vorinostat (SAHA); TYA‐018; CS1 (controlled‐release formulation of valproic acid); panobinostat	Supported in postinfarction remodeling, HFpEF and diastolic dysfunction, atherosclerotic plaque remodeling and stabilization, and PAH, with additional support from human vascular tissue and cell studies	Preclinical (animal models only) across cardiac, atherosclerotic, and pulmonary vascular models; Phase II ongoing in a CVD population (PAH)	Late‐phase oncology clinical trial experience is available for panobinostat.	Cytopenias, particularly thrombocytopenia and lymphopenia; diarrhea; asthenia/fatigue	[62, 230, 231, 232, 233, 234]
BET bromodomain inhibition	Acetyl‐lysine reading by BET proteins (BD2‐biased or pan‐BET)	Apabetalone (RVX‐208); (+)‐JQ1; molibresib	Supported in heart failure and vascular inflammatory remodeling, with additional support from human endothelial and monocyte studies and biomarker modulation in CVD populations; among chromatin‐reader targets, BET proteins currently have the most advanced cardiovascular clinical development.	Preclinical (animal models only) for disease‐modifying efficacy in heart failure and vascular inflammatory remodeling; Phase II and Phase III completed in CVD populations; pilot clinical study in PAH	Clinical development has reached late‐stage evaluation with apabetalone; broader BET inhibitors have mainly been developed in oncology.	Transaminase elevations and treatment discontinuation with apabetalone; thrombocytopenia and gastrointestinal toxicity are more prominent with broader BET inhibition.	[8, 235, 236, 237, 238, 239]
H3K27 methylation‐axis modulation (EZH2/JMJD3‐linked)	H3K27 methylation (EZH2‐mediated deposition) and H3K27 demethylation (JMJD3‐mediated removal)	GSK126; GSK‐J4; tazemetostat	Supported in myocardial fibrosis, postinfarction fibrotic remodeling, and cardiomyocyte hypertrophy, implicating the H3K27 axis in fibroblast activation and pathological hypertrophic gene programs during cardiac remodeling	Preclinical (animal models only)	Tazemetostat has Phase I/II oncology clinical trial experience; GSK126 and GSK‐J4 remain preclinical/tool compounds.	Asthenia/fatigue; nausea; anemia/cytopenias, including thrombocytopenia at higher doses	[215, 240, 241, 242, 243]
DOT1L inhibition	H3K79 methylation (predominantly H3K79me2/H3K79me3)	Pinometostat (EPZ‐5676)	Supported in myocardial fibrosis and postinfarction fibrotic remodeling; DOT1L inhibition attenuates cardiac fibroblast activation, ECM deposition, and cardiac dysfunction, supporting H3K79 methylation as a fibrosis‐associated chromatin target in cardiac remodeling.	Preclinical (animal models only)	Pinometostat has Phase I oncology clinical trial experience.	Fatigue; nausea; constipation; febrile neutropenia/cytopenias	[197, 198, 244]
Histone acetyltransferase inhibition (p300/CBP‐linked)	Histone acetylation (writer inhibition)	L002; C646	Supported in hypertension‐associated cardiac hypertrophy, myocardial fibrosis, diastolic dysfunction, and coronary microvascular dysfunction; p300 inhibition attenuates fibroblast activation, histone acetylation, and pathological remodeling, supporting writer‐directed targeting in cardiac remodeling.	Preclinical (animal models only)	Preclinical/tool‐compound stage; no established clinical development program.	No established human safety dataset; selectivity and translational PD validation remain limiting.	[104, 179, 245]

*Note*: Table 2 summarizes selected chromatin‐targeting strategies from Table 1 together with their targeted histone modification, cardiovascular application prospects/theoretical basis, CVD clinical development status, representative pharmacologic agents or interventions, and major safety or translational considerations. “CVD Clinical Development Status” refers specifically to cardiovascular development status and is standardized as preclinical (animal models only), preclinical (animal models with supportive human data), or completed/early clinical evaluation in a cardiovascular population. “Overall Clinical Development Status” refers to the broader noncardiovascular or general drug‐development context. Dose‐limiting or notable toxicities were standardized at a comparable level of detail across rows.

Abbreviations: BD2, bromodomain 2; BET, bromodomain and extraterminal domain; CBP, CREB‐binding protein; CVD, cardiovascular disease; DOT1L, disruptor of telomeric silencing 1‐like; ECM, extracellular matrix; EZH2, enhancer of zeste homolog 2; HDAC, histone deacetylase; HFpEF, heart failure with preserved ejection fraction; JMJD3, Jumonji domain‐containing protein 3; PAH, pulmonary arterial hypertension; PD, pharmacodynamic; SAHA, suberoylanilide hydroxamic acid.

### 5.1 HDAC Inhibitors

HDAC inhibition is among the best‐developed pharmacological routes for modulating acetylation‐linked programs in CVD [246, 247]. Tool compounds used in cardiovascular studies are often Class I‐focused and frequently also inhibit HDAC6, whereas strong catalytic targeting of Class IIa enzymes is less common; isoform attribution, therefore, matters because perturbation of distinct HDAC classes can yield divergent remodeling outcomes [246, 247]. In practice, HDAC inhibitors differ in isoform coverage and selectivity profiles [246].

Across pressure overload and neurohormonal stimulation models, HDAC inhibition reduces cardiomyocyte hypertrophy, blunts fetal gene reactivation, and limits interstitial fibrosis, with additional evidence for suppression of atrial fibrotic remodeling in preclinical models [246, 248, 249]. HDAC inhibitors also act outside cardiomyocytes, including direct antifibrotic effects in cardiac fibroblasts and immunomodulatory effects that can shape inflammatory remodeling [249]. In Ang II‐driven remodeling, Class I selective HDAC inhibition with MGCD0103 (mocetinostat) suppresses cardiac fibrosis by inhibiting fibroblast cell‐cycle progression and blocking fibrocyte differentiation [250]. These effects are not confined to histone substrates, because HDAC inhibition can also alter nonhistone acetylation and kinase signaling linked to fibrotic output [249, 251].

HDAC inhibition can be protective in acute cardiac injury models [252]. In MI/R, distinct HDAC inhibitors reduce infarct size and alter hypoxia‐responsive transcription, with the clearest benefit when dosing occurs early after reperfusion [252]. After MI, HDAC inhibition has been linked to macrophage polarization toward reparative phenotypes [253] and, in separate models, to c‐kit–dependent myocardial repair with improved ventricular function [254]. These findings indicate that dosing time is likely to be an important determinant of effect in acute reperfusion settings [252]. In oncology, HDAC inhibitors have been linked to electrophysiological adverse effects, including QT/QTc prolongation and arrhythmias, although exposure–QTc relationships are not uniformly observed across agents [255, 256, 257]. These liabilities make careful dose and exposure control essential in cardiovascular translation [255, 258]. Against this safety backdrop, HFpEF is an important translational setting for HDAC targeting, particularly in models that incorporate metabolic stress [231]. In a feline pressure‐overload model, HDAC inhibition improved cardiopulmonary structure and function and altered myocardial metabolic indices [259]. In rodent HFpEF models, HDAC inhibition reversed established diastolic dysfunction and blocked ECM expansion and LV stiffening while preserving ejection fraction [260]. These studies support therapeutic strategies aimed at reversing established disease rather than prevention alone [259, 260]. HDAC6 is of interest in this context because it deacetylates cytoskeletal and chaperone substrates and modulates stress‐linked pathways relevant to diastolic function [231]. In mice, selective HDAC6 inhibition reversed HFpEF‐like phenotypes, required HDAC6 for full efficacy, and showed added benefit with a sodium–glucose cotransporter 2 inhibitor [231].

Preclinical studies place HDAC inhibition among the most pharmacologically mature strategies for modulating acetylation‐linked programs in CVD, with consistent antihypertrophic and antifibrotic effects across remodeling models [246, 249]. Notably, this evidence base now extends to HFpEF, where HDAC targeting has been reported to reverse established diastolic dysfunction in both large‐ and small‐animal settings [231, 259, 260]. Reported electrophysiological liabilities with several clinical HDAC inhibitors, particularly QT‐interval prolongation and arrhythmias at higher systemic exposures, underscore the need for exposure‐aware dosing and structured ECG and electrolyte monitoring during clinical translation [255, 258].

### 5.2 BET Protein Inhibitors

In the heart, the BET bromodomain inhibitor (+)‐JQ1 is frequently used to probe BET‐dependent transcription in vivo. When started early after TAC, JQ1 preserves LV systolic function and limits chamber dilation and wall thickening, with reduced cardiomegaly and pulmonary congestion [8]. In therapeutic protocols, JQ1 remains active when initiated after HF is established during prolonged pressure overload and after a massive anterior wall MI, alongside preferential suppression of stress‐inducible ECM and innate immune transcriptional programs [261].

In diabetes‐accelerated AS, JQ1 reduced thoracic aortic plaque area in streptozotocin‐treated ApoE^−/−^ mice [262]. In primary VSMCs, JQ1 suppressed high glucose‐induced proliferation and migration and implicated a peptidyl‐prolyl cis–trans isomerase NIMA‐interacting 1 (Pin1)–BRD4 axis [262]. Apabetalone (RVX‐208) provides bromodomain 2 (BD2)‐biased BET inhibition. It is an oral BET bromodomain inhibitor with preferential engagement of BD2 over BD1 in biochemical and cellular assays [263, 264]. BD2‐biased inhibition offers a pharmacological alternative to pan BET probes such as (+)‐JQ1 for defining reader‐dependent transcriptional control in cardiovascular‐relevant contexts.

Pulmonary arterial hypertension (PAH) has also been used to test BET reader function in vivo. BRD4 is upregulated in lungs and pulmonary arteries from patients with PAH, and BRD4 inhibition with JQ1 or BRD4‐targeting small interfering RNA (siBRD4) improves pulmonary hemodynamics and attenuates vascular remodeling in the Sugen hypoxia rat model [265]. A subsequent multicenter randomized preclinical study tested RVX‐208 (apabetalone) and reported hemodynamic and remodeling benefits across several rodent PAH models, with parallel phenotyping of right ventricular responses [266].

BET bromodomain inhibition attenuates stress‐responsive BET‐dependent transcription and reduces inflammatory and fibrotic remodeling. Effects are reproduced across pressure overload, MI, and vascular remodeling models [8, 261, 262, 265, 266]. Other clinical‐stage BET inhibitors, such as molibresib, have faced dose‐limiting thrombocytopenia in oncology settings [239, 267]. This contrasts with the BD2‐biased apabetalone, which has accrued Phase II/III cardiovascular experience [236, 237].

### 5.3 HMT/DMT Inhibitors

Compared with acetylation‐ and BET‐directed approaches, pharmacological interrogation of lysine methylation in CVD remains less mature and is often context dependent across cardiac cell compartments [268, 269]. For now, therapeutic hypotheses rest mainly on well‐validated preclinical models, ideally paired with cell‐type‐resolved genetic perturbation and in vivo PD readouts that demonstrate target engagement and on‐target effects, rather than relying on downstream pathway signatures alone [270].

H3K27 methylation has been interrogated with small‐molecule probes that target both the EZH2 writer and the JMJD3 eraser. In an isoproterenol‐driven remodeling model, the EZH2 inhibitor GSK126 improved cardiac remodeling and reduced myocardial fibrosis in vivo, alongside rescue of fibroblast activation in complementary in vitro assays [240]. In postinfarction remodeling, pharmacological inhibition of JMJD3 with GSK‐J1 attenuated cardiac fibrosis and improved cardiac function, with supporting fibroblast data showing reduced ECM gene output in Ang II‐activated cardiac fibroblasts [215]. In cardiomyocytes, the JMJD3 tool inhibitor GSK‐J4 suppressed isoproterenol‐induced hypertrophy and blunted the induction of fetal‐gene markers, including atrial natriuretic factor (ANF), BNP, and β‐myosin heavy chain [241]. Available studies indicate that manipulating H3K27 methylation can modulate both fibroblast‐driven fibrosis and cardiomyocyte hypertrophic programs. Translation will require cell‐type‐resolved target validation and in vivo confirmation of H3K27 mark engagement under chronic dosing.

In pressure overload and β‐adrenergic stress, cardiac DOT1L–H3K79me2 signaling is induced. Cardiomyocyte DOT1L deletion blunts adverse remodeling and lowers fetal‐gene output [149]. Pharmacological DOT1L inhibition with SGC0946 mitigates isoproterenol‐induced hypertrophy and reduces myocardial H3K79me2 and ANP [149]. In leukemia, pinometostat delivered by continuous infusion achieves measurable H3K79me2 suppression in patients [244]. For cardiovascular use, dose and exposure should be guided by in vivo on‐mark suppression of cardiac H3K79me2 under chronic dosing, not by downstream phenotype alone.

Not all histone methylation enzymes are suitable inhibitory targets in the heart, because some support stress adaptation. In an isoproterenol model, nuclear receptor‐binding SET domain protein 3 (NSD3) expression fell with prohypertrophic stimulation, and cardiomyocyte NSD3 overexpression attenuated hypertrophy, with ANF‐promoter chromatin changes and a proposed NSD3–BRD4‐linked mechanism [271]. Cardiomyocyte KMT2D/MLL4 has also been linked to adaptive responses during pressure overload, and its cardiomyocyte‐specific deletion exacerbated decompensation phenotypes [272]. In CVD, methylation‐enzyme druggability must be judged by cell‐specific function, not by enzyme class alone.

Preclinical studies indicate that selected H3K27 and H3K79 nodes can be manipulated to attenuate fibrotic and hypertrophic remodeling, although tool specificity and context dependence remain practical constraints for interpretation and translation [149, 215, 241]. Oncology experience with pinometostat illustrates that sustained systemic exposure can deliver measurable on‐mark H3K79me2 suppression in patients, a useful precedent for target‐engagement strategy design [244]. Similarly, the United States Food and Drug Administration‐approved EZH2 inhibitor tazemetostat demonstrates manageable safety profiles in sarcoma and lymphoma trials, though risks of secondary myeloid neoplasms and cytopenias require vigilance [243, 273]. Moving toward cardiovascular translation will require cell‐type‐resolved target validation and on‐mark PDs that confirm in vivo engagement of the relevant histone methylation mark under chronic dosing [270].

### 5.4 Targeting Lactylation, Crotonylation, and Related Metabolite‐Derived Lysine Acylations

Metabolite‐derived histone lysine acylations, including lactylation and crotonylation, provide a route by which metabolic state can be coupled to chromatin regulation. Kla was described in 2019 as a lactate‐linked modification that can promote gene transcription [7]. In cardiovascular remodeling, therapeutic leverage over these acyl marks remains largely indirect [157, 274]. Therapeutic translation will require mark‐selective chemical tools and quantitative, cell‐resolved assays that can demonstrate on‐mark engagement in vivo.

For Kla, the most compelling evidence comes from hypertrophic stress models in which myocardial lactate accumulation coincides with increased Kla and can be functionally opposed by limiting lactate production [158]. In TAC and cardiomyocyte hypertrophy settings, pan‐Kla and H3K18la rise most consistently, whereas additional lactylation sites can also change in cardiomyocyte hypertrophy models, and inhibiting glycolysis, LDH activity, or LDHA reduces lactylation readouts alongside attenuation of pathological growth phenotypes [158, 275]. When the intervention is metabolic, the primary readout should be a matched measurement of myocardial lactate and site‐resolved lactylation in the same tissue and cell compartment, rather than downstream pathway signatures.

The functional impact of Kla appears to be context and cell‐type‐dependent across cardiovascular compartments [76, 140]. After MI, monocyte–macrophage lineage cells show glycolytic rewiring with MCT1‐mediated lactate transport that promotes Kla and facilitates reparative gene transcription [76]. In AS‐relevant endothelial settings, H3K18la increases and contributes to EndMT programs through an ASF1A‐ and p300‐linked mechanism [140]. Collectively, these findings favor timing‐ and cell‐specific strategies over uniform systemic suppression of lactylation [76, 140].

Crotonylation highlights that acyl‐mark manipulation can be directionally distinct across cardiovascular contexts. Sodium crotonate increases overall protein lysine crotonylation and limits cardiomyocyte injury after ischemia–reperfusion, with dominant crotonylation changes reported in extranuclear protein compartments rather than a histone‐restricted program [44]. ECHS1 regulates crotonyl‐CoA availability and constrains histone H3K18cr and H2BK12cr during hypertrophic signaling, while crotonate supplementation increases histone Kcr and promotes cardiomyocyte hypertrophy [159].

Lactylation and crotonylation are, in principle, addressable through installing enzymes, removing enzymes, and acyl‐lysine binding modules, but cardiovascular validation remains limited. Class I HDACs, particularly HDAC1 and HDAC3, show delactylase activity in cells, and HDAC1–3 are active in biochemical assays [276]. Class I HDACs also act as major histone decrotonylases, and SIRT1 to SIRT3 show decrotonylase activity, indicating that deacetylase families can modulate more than one acyl mark [277, 278]. ACSS2 has been identified as a lactyl‐CoA synthetase, and when coupled to KAT2A can support histone lactylation in mammalian cells [279]. HBO1 has also been shown to catalyze histone lactylation, with strong evidence for H3K9la regulation in mammalian cells [280]. YEATS domains preferentially bind crotonyl‐lysine, and YEATS inhibitors have been developed in oncology, but cardiovascular relevance remains untested [281, 282].

Overall, lactylation and crotonylation connect metabolic state to chromatin regulation in cardiovascular remodeling, but efforts to modulate these marks therapeutically remain largely preclinical. Near‐term progress will depend on pairing metabolic perturbation with direct, cell‐resolved quantification of the relevant metabolite pool and the corresponding histone acyl mark in the same cardiovascular compartment. This distinction matters because lactylation aligns with hypertrophic programs in pressure‐overloaded cardiomyocytes yet supports reparative transcription in infarct macrophages, and increasing protein crotonylation has been reported to limit cardiomyocyte injury after ischemia–reperfusion [7, 44, 76, 158]. In the longer term, translation will depend on mark‐selective probes and compartment‐focused delivery, supported by in vivo readouts that report acyl‐mark engagement directly.

### 5.5 Gene Editing and Epigenetic Reprogramming

Alongside small‐molecule epigenetic modulators, gene and epigenome engineering enable programmable, locus‐specific perturbation of cis‐regulatory elements, converting epigenomic associations into causal tests of regulatory function [283, 284, 285]. Catalytically inactive Cas9 (dCas9) can be fused to chromatin‐modifying effectors, such as p300 or LSD1, to deposit or erase enhancer‐ and promoter‐associated histone features at selected loci and directly test whether local chromatin rewriting is sufficient to alter transcriptional output [284, 285].

Among CRISPR (clustered regularly interspaced short palindromic repeats)‐derived epigenome editors, the dCas9–p300 fusion increases H3K27ac at targeted promoters and distal enhancers and activates endogenous gene transcription at the chosen locus [284]. Programmable repression can be achieved with CRISPR interference, in which nuclease‐dead dCas9 fused to Krüppel‐associated box (KRAB) domains represses transcription without generating DNA double‐strand breaks and recruits repressive chromatin‐modifying complexes to the targeted site [283, 286]. In inducible in vivo settings, dCas9–KRAB targeting can be accompanied by targeted repressive chromatin‐state changes at the edited locus [287]. Enhancers can also be edited directly, as dCas9–LSD1 can inactivate selected enhancers in their native chromatin context with loss of enhancer‐associated marks, including H3K4me2 and H3K27ac at the targeted region [285]. Inducible, tissue‐restricted transgenic editor systems further establish organ‐level feasibility while underscoring practical constraints for cardiovascular deployment, particularly construct payload and the limits of viral vector packaging and delivery [287, 288]. Epigenetic memory editors such as CRISPRoff extend this framework by showing that transient editor exposure can yield durable gene silencing, a potentially useful property for chronic indications if editor delivery and activity can be appropriately controlled [289].

Cardiac reprogramming provides a relevant application context in which fate conversion is coupled to chromatin state changes. During fibroblast‐to‐cardiomyocyte conversion, extensive epigenetic repatterning accompanies de‐repression of cardiogenic gene programs [290]. Proof‐of‐concept studies showed that enforced expression of cardiogenic transcription factors can convert fibroblasts into induced cardiomyocyte‐like cells [291]. In MI models, local delivery of transcription factor cocktails generated induced cardiomyocyte‐like cells in injured myocardium and was accompanied by improvements in ventricular function or remodeling [292, 293]. MicroRNA‐based protocols have also been reported to support in vivo conversion after infarction [294]. Across direct cardiac reprogramming studies, however, efficiency remains limited, and maturation is often incomplete [290, 295]. CRISPR‐based transcriptional activation, termed CRISPR activation (CRISPRa), has likewise been incorporated into reprogramming strategies by activating endogenous cardiac regulators [296, 297]. CRISPRa activation of developmental regulators produced cardiovascular progenitor‐like cells that improved contractile function and reduced scar burden after implantation into postinfarction mouse hearts [296]. A 2024 study further showed that CRISPRa activation of endogenous GATA4, combined with exogenous MEF2C and TBX5, can generate induced cardiomyocytes, and that CRISPRa‐mediated activation of MEF2C and TBX5 is less effective than endogenous GATA4 activation, consistent with locus‐dependent chromatin constraints [297].

Clinical translation of cardiovascular epigenome editing will depend on delivery platforms that enable tissue‐restricted delivery and practical control of editor exposure. Inducible dCas9–p300 expression in vivo can cause guide RNA‐independent, genome‐wide shifts in H3K27ac and transcription [287]. This raises concern about sustained editor activity and supports limiting exposure duration to reduce cumulative locus‐independent chromatin effects. Transgene silencing during lineage transitions represents an additional constraint, since dCas9–VPR can be active in pluripotent cells but becomes silenced during differentiation toward cardiomyocyte and endothelial fates, with evidence implicating promoter methylation [298]. Recent in vivo genome editing trials provide practical reference points for systemic delivery and safety monitoring in cardiovascular‐related settings, including first‐in‐human CRISPR/Cas9 therapy for transthyretin amyloidosis [299]. Base editing of proprotein convertase subtilisin/kexin Type 9 further shows that a single course can yield durable lowering of a cardiovascular risk factor in nonhuman primates, even though it targets DNA sequence rather than histone marks [300].

### 5.6 Clinical Trials and Translational Opportunities

Clinical translation of chromatin‐directed interventions in CVD is still limited. Intervention studies nonetheless provide a practical basis for target prioritization and translational assessment. They help separate targets supported by reproducible in vivo efficacy and measurable biological readouts from those constrained by context dependence or safety liabilities [5, 255]. We summarize preclinical intervention evidence that couples disease‐relevant phenotypes to on‐target modulation of the intended chromatin pathway in cardiovascular tissues [8, 149], and then integrate the available human biomarker and interventional data that help guide clinical prioritization [81, 237].

#### 5.6.1 Preclinical and Early Translational Evidence Across Histone PTMs

Despite expanding preclinical evidence, clinical translation of chromatin‐directed interventions in CVD remains limited. In vivo intervention studies and translational analyses provide the clearest evidence that perturbing histone PTM‐linked pathways can modify cardiovascular remodeling, while also highlighting practical translational considerations such as target engagement, model dependence, and treatment feasibility [8, 55, 104, 149]. Across pressure overload, vascular disease, and cardiomyopathy/HF remodeling, several studies also include human tissue correlates or mechanistic readouts that support pathway selection beyond associative profiling [156, 158, 187].

In murine TAC, BET bromodomain inhibition with (+)‐JQ1 reduced LV hypertrophy and fibrosis and preserved systolic function [8]. The H3K79 methyltransferase DOT1L also influenced remodeling, as cardiomyocyte‐specific DOT1L deletion improved ejection fraction and fractional shortening after TAC and blunted reactivation of fetal‐gene programs [149]. Histone phosphorylation intersects stress signaling, as CaMKIIδ‐dependent H3S10ph was required for the hypertrophic response and for transcriptional activation of hypertrophic genes under hemodynamic stress [154]. In hypertensive remodeling models, p300 acetyltransferase inhibition (L002 or C646) reduced hypertrophy and fibrosis and ameliorated diastolic dysfunction, supporting in vivo targeting of acetylation writers [104].

In pressure‐overload models, lactate‐associated Kla has been implicated in pathological cardiac hypertrophy [158, 275]. H3K18la and pan‐Kla increased in TAC hearts and failing human myocardium, and oxamate treatment or cardiomyocyte‐specific LDHA deletion attenuated TAC remodeling; in parallel, oxamate reduced H3K18la enrichment at the TGFB2 promoter and blunted TGFB2 induction [158].

Related evidence extends to hypertrophic and vascular disease models that couple metabolite availability to chromatin regulation. In Ang II‐stressed hearts and cardiomyocytes, ECHS1 deficiency increased H3K18cr and H2BK12cr and promoted hypertrophic growth together with induction of hypertrophic gene expression [159]. Crotonate supplementation was used to modulate crotonyl‐CoA availability and test crotonylation‐dependent effects [159]. In ApoE^−/−^ AS, endothelial ASF1A loss attenuated EndMT and alleviated AS development [140]. This effect coincided with reduced SNAI1 transcription, supporting an H3K18la‐associated program as a contributor to lesion biology in vivo [140]. In injury‐driven neointimal remodeling, SMC PRMT5 silencing and pharmacologic PRMT5 inhibition (EPZ015666) reduced neointimal hyperplasia; complementary in vitro data showed preservation of contractile SMC marker programs, and PRMT5 upregulation was also documented in human atherosclerotic lesions [187]. VSMC TRAP1 deficiency and TRAP1‐targeting approaches reduced AS severity together with VSMC senescence and SASP signatures linked to H4K12la [191]. KDM1A inhibition further reduced atherosclerotic lesion burden and attenuated vascular oxidative stress and inflammatory signaling [74].

PAD inhibition with Cl‐amidine reduced H3Cit‐positive NETs, lesion area, and delayed carotid thrombosis, and deoxyribonuclease I treatment reduced plaque NET content and plaque macrophage area during regression [55, 79]. Notably, myeloid Hdac3 deletion increased plaque collagen and fibrous‐cap thickness but also increased lesion size [62]. Lesion burden was reduced in ApoE^−/−^ models with macrophage monocarboxylate transporter 4 (MCT4) depletion, which was accompanied by activation of reparative macrophage programs [190].

State‐resolved in vivo mapping after MI showed H3K18la enrichment at reparative loci in infarct‐associated monocyte and macrophage states, consistent with a role for this acyl mark in wound‐healing transcriptional programs [76]. In HFpEF‐like remodeling, selective HDAC6 inhibition with TYA‐018 reduced inflammatory signaling and improved diastolic dysfunction and stiffness‐related phenotypes in mice [231]. Beyond acetylation‐linked approaches, TRIM35‐driven H2BK120ub was associated with heart‐failure‐related transcriptional remodeling through increased p53‐target accessibility and transcription in cardiomyopathy models, with elevated TRIM35 and H2Bub1 also observed in human DCM [156].

In vivo support is not equally developed across histone PTM‐linked pathways. The strongest cases are those in which phenotypic benefit is accompanied by direct target‐engagement readouts and corresponding human data [8, 55, 149, 158, 187].

#### 5.6.2 Toward Clinical Relevance Through Human and Interventional Studies

Clinical quantification is supported by associations between circulating H3Cit–DNA complexes and 1‐year MACE after STEMI [81]. However, this finding was not reproduced in another STEMI cohort, in which H3Cit–DNA did not predict 30‐day MACE or mortality, underscoring the need for further validation before clinical use [301]. Culprit lesion data add biological context. Thrombus NET burden and endogenous DNase activity relate to ST‐segment resolution and infarct size [302]. NET detection in aspirated coronary thrombi relates to early post‐STEMI MACE, particularly within the first 30 days [303]. Interventional studies then test whether BET‐directed modulation can be administered safely enough for cardiovascular use while retaining measurable biological activity [236, 237].

In this context, clinical trial experience is most developed for BET inhibition with apabetalone (RVX‐208). In patients with angiographic coronary disease and low HDL‐C, ASSURE did not show incremental intravascular ultrasound (IVUS) plaque regression and reported hepatic transaminase elevations [236]. In high‐risk patients after acute coronary syndrome (ACS), BETonMACE likewise did not significantly reduce MACE and again reported transaminase elevations [237]. Against these neutral primary outcomes, Phase II biomarker analyses reported changes in circulating mediators linked to vascular inflammation and plaque destabilization, while IVUS subanalysis suggested favorable modulation of plaque features associated with vulnerability [235, 304]. A pooled analysis of ASSERT, ASSURE, and SUSTAIN suggested fewer MACE, but the component trials were short and not powered for outcomes [305].

The apabetalone program underscores the importance of matching indication and endpoint selection to the biology of BET inhibition, with PD readouts incorporated where feasible [236, 237]. Prespecified BETonMACE analyses remained hypothesis‐generating, with nominal signals for reduced HF hospitalization burden and effect estimates favoring apabetalone in the CKD subgroup [9, 306]. An open‐label PAH pilot further addressed feasibility outside atherosclerotic settings [238].

For HDAC inhibition, a gap remains between extensive preclinical evidence and cardiovascular‐specific clinical testing [5]. CS1, a controlled‐release formulation of valproic acid, provides an early example of cardiovascular‐oriented clinical evaluation [233]. Earlier human pharmacology studies using valproic acid also reported quantifiable vascular fibrinolytic readouts, including reduced plasminogen activator inhibitor‐1 levels in healthy volunteers and reduced exhaustion of stimulated tissue plasminogen activator release capacity in subjects with vascular disease, providing a precedent for PD‐anchored translation in this class [307, 308]. The same issues recur at the translational stage. Biomarker strategies for tracking target‐proximal chromatin effects in vivo remain underdeveloped, and human cardiac epigenomic maps are still needed to refine disease‐relevant regulatory programs for target prioritization [309, 310, 311].

Much of the safety experience for HDAC inhibitors comes from oncology, where systemic exposures can be justified despite clinically important hematologic toxicities and QT/arrhythmic liability, whereas in CVD the same safety profile may become rate‐limiting because treatment is typically chronic and the margin for sustained dosing is narrower [255]. This is particularly relevant for QT risk, because polypharmacy, drug–drug interactions, and electrolyte disturbances, especially hypokalemia and hypomagnesemia, increase susceptibility to torsades de pointes [312]. High‐grade cytopenias and infection‐related complications, including febrile neutropenia, can also become dose‐limiting in panobinostat‐containing oncology regimens [313]. These constraints support development strategies that incorporate baseline risk assessment, protocolized ECG and electrolyte monitoring, and prespecified patient‐selection criteria [255, 312].

In CVD, the translational issue is now less whether chromatin‐directed interventions can modify disease‐relevant phenotypes than which targets can be advanced with interpretable PD readouts and an acceptable safety margin. The strongest candidates are those supported by reproducible in vivo efficacy and at least some concordant human data. NET‐related biomarkers after STEMI and the apabetalone program illustrate both the value and the limits of current human signals, while HDAC‐directed approaches further indicate that broader clinical development will require tractable in vivo markers of on‐target chromatin modulation, disease‐relevant human epigenomic context, and safety frameworks compatible with chronic treatment [8, 81, 149, 237, 255, 301, 306, 307, 308, 309, 310, 311, 312].

## 6 Emerging Technologies and Future Research Directions

Work in cardiovascular epigenetics is increasingly shaped by methods that resolve cell states, tissue context, and locus‐specific regulation, together with tools that quantify chromatin modulation in vivo. This section reviews technologies for cell‐resolved mapping, causal inference, and translational PD readouts.

### 6.1 Single‐Cell and Multiomics Technologies

Technical caveats should be considered when reconciling conflicting histone PTM reports across platforms and laboratories [314, 315]. Most locus‐resolved histone PTM profiling remains antibody dependent, and widely used commercial reagents show substantial variability in performance and off‐target binding across related epitopes and experimental contexts [316, 317, 318]. Peptide array benchmarking and community resources such as the Histone Antibody Specificity Database support reagent pre‐screening, but assay‐specific validation remains essential because neighboring PTMs can alter epitope recognition and generate technical rather than biological differences in apparent signal [314, 317, 319]. Assay choice also affects comparability across studies. Cleavage under targets and release using nuclease (CUT&RUN) and cleavage under targets and tagmentation (CUT&Tag) reduce background and improve signal‐to‐noise relative to conventional chromatin immunoprecipitation sequencing (ChIP‐seq), and they enable low‐input profiling [315, 320]. Direct comparisons across platforms, therefore, require careful normalization and like‐for‐like analytical handling [320]. Site‐resolved histone mark quantification by mass spectrometry provides an orthogonal readout [321, 322]. However, low stoichiometry, isobaric and combinatorial modifications, and preparation‐related bias necessitate standardized workflows and normalization for robust comparisons [321, 322]. These considerations are particularly relevant for lysine acylations. Cross‐reactivity of nonacetyl acylation antibodies with acetylation and related epitopes has been documented, and orthogonal confirmation is often needed for mark assignment [323, 324]. When reports conflict, confidence is greatest when antibody performance is documented [314, 325]. Key findings should therefore be corroborated by orthogonal assays, for example, by pairing locus‐resolved profiling with site‐resolved quantification where matched material is available [321, 324].

Bulk epigenomic profiling averages signals across cardiomyocytes, fibroblasts, endothelial, and immune cells. It therefore under‐resolves cell‐type‐ and cell‐state‐specific cis‐regulatory programs in cardiovascular remodeling. Single‐nucleus atlases link chromatin accessibility to cell identity through integration with transcriptomic information and are beginning to map regulatory programs in adult human heart and coronary artery tissue [326, 327]. These resources can also nominate likely effector cell types for cardiovascular risk variants using cell‐type‐restricted cis‐regulatory landscapes [326, 327]. Multimodal single‐cell profiling adds orthogonal layers that sharpen state annotation. In atherosclerotic lesions, approaches that pair transcriptomes with surface‐protein readouts, for example, cellular indexing of transcriptomes and epitopes by sequencing (CITE‐seq), improve immune and stromal state assignment and support comparisons across clinically defined lesion groups anchored to defined cell populations [328].

Beyond accessibility and transcriptome‐centered modalities, direct single‐cell profiling of histone PTMs is now practical and increasingly scalable. Single‐cell CUT&Tag (scCUT&Tag) adapts CUT&Tag to high‐throughput single‐cell workflows and enables profiling of histone marks and chromatin‐bound factors with low background [329, 330]. ScCUT&Tag‐pro further increases scale and extends single‐cell chromatin profiling into multimodal settings, including joint measurement of protein–DNA interactions and surface proteins [331]. Multiomic assays that couple chromatin state to transcription, including CoTECH (coassay of transcriptome and epigenome) and Paired‐Tag, provide paired measurements of histone marks or chromatin occupancy together with RNA from the same cell [332, 333]. Droplet‐based Paired‐Tag improves throughput and highlights antibody performance as an important determinant of single‐cell signal quality [334]. TACIT (targeted chromatin integration tagging) increases per‐cell genome coverage for histone‐modification profiling [335]. scEpi2‐seq jointly profiles CpG methylation and H3K27me3 within the same single cells [336]. Despite increasing scalability, sparsity remains a central limitation. Per‐cell read depth is often only a few hundred to a few thousand reads, and analytical choices do not transfer directly from single‐cell ATAC sequencing or scRNA‐seq. Recent benchmarks provide single‐cell histone PTM‐specific guidance for study design and downstream computation [337].

### 6.2 Spatial Epigenomics

Single‐cell assays generally require tissue dissociation and therefore remove spatial context [338]. Spatially resolved studies of MI and human atherosclerotic plaques show that remodeling programs are regionally patterned, including infarct border zones and spatially distinct plaque regions [339, 340, 341]. Spatial epigenomics aims to assign chromatin features to histological coordinates in tissue sections.

Sequencing‐based platforms now support genome‐wide spatial chromatin profiling. Spatial chromatin accessibility can be captured using microfluidic barcoding in a spatial assay for transposase‐accessible chromatin sequencing (spatial ATAC‐seq) [338] or solid‐phase capture approaches [342]. sciMAP‐ATAC (single‐cell combinatorial indexing–based micromap ATAC) uses position‐annotated microbiopsies coupled to combinatorial‐indexing single‐cell ATAC‐seq, with spatial granularity defined by the sampling scheme [343]. For histone marks, Spatial‐CUT&Tag profiles histone modifications in situ and can derive single‐cell epigenomes in situ by identifying 20 µm pixels containing only one nucleus using immunofluorescence imaging [344]. Spatial epigenome–transcriptome coprofiling measures histone marks and gene expression on the same section, including H3K27me3, H3K27ac, and H3K4me3 [345]. Slide‐tags barcodes nuclei in intact sections and enables downstream processing with standard single‐nucleus workflows, including single‐nucleus RNA sequencing and single‐nucleus ATAC sequencing [346].

In CVD, spatially anchored multiomics is being used across regionally organized injury and tissue niches. Studies integrate spatial transcriptomics with single‐nucleus transcriptomes and chromatin accessibility maps [339]. Similar strategies have been applied to the adult human heart, delineating cellular niches across anatomical regions using single‐cell and spatial transcriptomic data [347]. In human atherosclerotic plaques, spatial transcriptomic studies localize site‐specific inflammatory and matrix‐remodeling programs to rupture‐prone or unstable regions [340, 341]. Spatial histone profiling provides a direct measurement of chromatin state within these microenvironments, including via Spatial‐CUT&Tag [344] and spatial CUT&Tag RNA‐seq [345]. Emerging multimodal platforms can combine histone marks with chromatin accessibility, transcriptome, and protein panels in the same section [348]. Key constraints include platform‐dependent differences in measurement area and throughput, tissue‐specific optimization of sample handling, and robust segmentation and cross‐modality integration in relation to tissue morphology [349].

### 6.3 Epigenetic Biomarkers for Diagnosis

A translational constraint is that disease‐relevant chromatin states are established in cardiovascular tissues, whereas routine clinical sampling is usually limited to blood. Human cardiac H3K27ac maps provide a tissue reference that links regulatory element activity to myocardial remodeling programs and places noncoding risk loci in a mechanistically plausible context [310, 311].

In blood, DNA methylation–based risk scores and classifiers remain the most developed blood‐based epigenetic readouts for coronary heart disease risk modeling and case classification. Their portability depends on external validation across cohorts and on models that account for conventional clinical risk factors [350, 351]. Array‐derived markers can be translated into scalable targeted assays, including methylation‐sensitive digital PCR, which supports clinical implementation [351].

Tissue‐of‐origin liquid biopsy is more proximate to diagnosis in acute injury. Cardiomyocyte‐selective cell‐free DNA (cfDNA) methylation markers quantify cardiomyocyte death kinetics and can complement established protein biomarkers [352]. This approach has been extended to MI and myocardial injury cohorts with droplet digital PCR‐compatible readouts [353]. cfDNA methylation profiles can also support ACS subtyping with independent‐cohort validation [354].

Histone‐linked blood biomarkers are supported by recent clinical and methodological work. In a small HF cohort, a plasma histone profile distinguished HFpEF from HF with reduced ejection fraction [355]. Cell‐free chromatin immunoprecipitation sequencing (cfChIP‐seq) applies chromatin immunoprecipitation to plasma nucleosomes and shows that circulating chromatin fragments retain histone modifications that report tissue‐linked transcriptional programs [356]. In heart transplant recipients, plasma cfChIP‐seq recovered pathway‐level signals and tissue‐of‐origin patterns relevant to heart transplant monitoring [357]. Fragmentomics provides an orthogonal readout, as cfDNA fragmentation features correlate with active histone marks, including H3K27ac and H3K4me3, enabling inference of histone‐modification‐associated signals from cfDNA sequencing without immunoprecipitation [309].

### 6.4 Challenges in Drug Delivery

Translation of epigenetic drugs to CVD often depends on cardiovascular‐selective exposure that is compatible with chronic dosing. Direct myocardial pharmacokinetic sampling and PD confirmation are rarely practical because endomyocardial biopsy is invasive and reserved for selected indications [358]. Many epigenetic regulators are broadly expressed, and most clinical experience comes from oncology, where systemic toxicities can be tolerated at exposures that are difficult to sustain in HF or AS. The HDAC inhibitor depsipeptide illustrates this constraint, as QTc prolongation has been documented in oncology trials [359], a liability that would complicate long‐term repurposing without compartment‐restricted exposure. Lesion‐targeted nanocarriers and in vivo screening of lipid nanoparticle (LNP) chemistries offer routes to improve cardiovascular selectivity [360, 361]. When biopsy is not feasible, martinostat‐based HDAC PET provides proof‐of‐principle for noninvasive biodistribution and occupancy assessment, although cardiovascular application remains preliminary [362, 363]. Cardiovascular validation remains limited, and links to myocardial histone‐mark modulation have not been established.

For small molecules, therapeutic‐index engineering must account for diffusion and perfusion heterogeneity in the remodeling myocardium. After reperfused MI, cardiac MRI can identify microvascular obstruction and regionally restricted perfusion [364, 365]. These regions associate with infarct healing and subsequent remodeling [364, 365]. After systemic dosing, delivery to atherosclerotic plaques is further constrained by endothelial access and local hemodynamics [366, 367]. High wall shear stress in stenotic segments makes vascular‐wall targeting and retention highly design dependent [366, 367]. Targeted nanomedicine can enrich payloads in lesion endothelium or plaque macrophages [368, 369], but performance remains sensitive to ligand orientation and presentation [369] and off‐target sequestration, including uptake in liver and spleen [368].

Delivery constraints are tighter for macromolecular modalities, including locus‐directed epigenome editing. Recombinant adeno‐associated virus (AAV) enables efficient in vivo gene transfer and shows preclinical cardiac tropism in mice, including cardiomyocyte transduction with AAV serotype 9 [370]. Cargo capacity limits remain a practical constraint [371, 372]. Neutralizing immunity can restrict dose and preclude redosing in chronic indications [371, 372]. Capsid engineering can improve cardiomyocyte tropism [371, 372], but clinical deployment still needs to balance dose requirements, immunogenicity, and long‐term safety [371, 372]. LNPs enable transient mRNA delivery [373]. Systemic delivery of modified mRNA has been demonstrated in injured myocardium, but myocardial access and endosomal escape remain limiting steps [373]. For CRISPR and dCas9 regulatory strategies, delivery and cell‐specific targeting remain major bottlenecks [371, 374].

### 6.5 Future Therapeutic Prospects

The next generation of cardiovascular epigenetic therapeutics will likely pivot from broad chromatin inhibition toward precision strategies guided by human myocardial regulatory maps that resolve disease‐linked enhancers and connect noncoding variation to remodeling programs [310, 311]. Progress will depend on prioritizing targets with human genetic and tissue epigenomic support. It will also depend on PD readouts that can be anchored to chromatin state in vivo, including plasma cfDNA fragmentation features that track active regulatory histone marks [309].

Within this human‐evidence framework, event‐driven pharmacology could expand the actionable space beyond catalytic inhibition. Targeted protein degradation enables event‐driven, sub‐stoichiometric depletion of chromatin‐bound epigenetic regulators, including writers, readers, and erasers [375, 376, 377]. Protein removal can attenuate both catalytic outputs and noncatalytic scaffolding functions without sustained active‐site occupancy [375, 376, 377]. This rationale is aligned with BRD4‐dependent, stress‐inducible enhancer and super‐enhancer programs that promote cardiac fibroblast activation and fibrotic remodeling [16]. It is also aligned with fibroblast‐selective cis‐regulatory nodes that can guide target discovery [378].

A second frontier is locus‐selective epigenome manipulation. CRISPR‐based epigenome editors show that chromatin marks can be installed at defined loci, exemplified by dCas9–p300‐mediated histone acetylation and dCas9‐tethered chromatin kinase–driven histone phosphorylation [24, 284]. In cardiovascular applications, clinical plausibility will depend on achieving tissue‐restricted delivery of these large effector constructs. It will also depend on meeting long‐term requirements for specificity, safety, and immunogenicity [379, 380].

Finally, the therapeutic scope should broaden as metabolite‐linked and other noncanonical histone PTMs become quantitatively measurable and experimentally tractable [381]. Current syntheses in HF, post‐MI remodeling, and endothelial dysfunction suggest that pharmacologic exposures, and in some settings lifestyle interventions, can modulate epigenetic programs relevant to cardiovascular pathophysiology [127, 382, 383]. Emerging work also implicates natural products as modulators of these pathways [383, 384]. Translation will require validated target–mark–phenotype chains and PD biomarkers that report in vivo target engagement. Overcoming these delivery and specificity barriers remains the final frontier in translating the promise of chromatin biology into disease‐modifying cardiovascular therapies. These advances narrow the gap between mechanistic chromatin biology and clinical translation by improving cell‐resolved mapping, causal attribution, and in vivo PD readouts. They also clarify the remaining priorities for cardiovascular epigenetic therapeutics.

## 7 Summary and Outlook

Histone PTMs link hemodynamic load, metabolic state, and inflammatory signaling to persistent changes in chromatin accessibility and gene expression in cardiovascular tissues. Acetylation and methylation remain the most extensively characterized marks, while phosphorylation, ubiquitination, lactylation, and crotonylation add further regulatory layers in cardiomyocytes [154, 156, 158, 159], vascular cells [140], and immune cells [7]. PTM writers, erasers, and readers then shape the magnitude and duration of injury‐responsive programs and are implicated in endothelial dysfunction under disturbed flow [130], fibroblast activation with ECM remodeling [16], VSMC phenotypic switching [123], and hypertrophic cardiomyocyte growth [149]. Functional output is context and stage‐dependent. Kla has been associated with hypertrophic and metabolic reprogramming under pressure overload [158], whereas in infarct‐associated monocyte‐to‐macrophage transitions, it aligns with reparative transcriptional programs [76]. In vascular endothelium exposed to AS‐relevant cues, H3K18la has been linked to EndMT through an ASF1A–p300‐dependent mechanism [140]. These examples illustrate how the same PTM class can map to distinct programs across cardiovascular cell states. Accordingly, causal inference in cardiovascular chromatin biology must account for cell state, locus specificity, and disease trajectory.

Reported effects often diverge not because the underlying biology is contradictory, but because the relevant effector cell compartment and sampling window differ across studies. For a given regulator, the key effector cell type can shift. The apparent direction of benefit then varies with where the perturbation is introduced and when phenotypes are assessed. In AS, HDAC3 has been linked to endothelial integrity under disturbed flow and lesion development [61]. Metabolite‐coupled PTMs add further context dependence. Similar metabolic shifts can be adaptive in one cell state yet pathogenic in another. Global suppression of a metabolite‐driven PTM may attenuate reparative programs together with maladaptive ones [7, 76, 140]. Pharmacological inference is constrained by on‐target and isoform attribution. Many HDAC inhibitors used in cardiovascular studies have broad class activity and extensive nonhistone substrates. This complicates isoform assignment and mechanistic interpretation [246, 247]. Technical factors also shape inference. Single‐cell assays often require tissue dissociation with loss of spatial information, and spatial platforms differ in capture area, throughput, and molecular depth [338, 349]. Cross‐study comparability improves when assay constraints are reported explicitly, and key claims are supported by orthogonal validation.

Several enabling technologies now make this workflow tractable. When a chromatin regulator is proposed as a therapeutic target, studies should define the cell population in which it is required, quantify relevant histone‐mark changes in vivo at disease‐relevant loci, and relate on‐target mark modulation to interpretable phenotypes along a defined disease trajectory. Parallel perturbation across candidate cell types, such as cardiomyocytes versus fibroblasts or endothelial versus myeloid cells, reduces misattribution from bulk‐tissue readouts. Human single‐nucleus and multimodal atlases map regulatory programs and nominate likely effector cell states for cardiovascular risk loci [326, 327]. scCUT&Tag and higher‐throughput variants enable histone‐mark profiling at scale with low input [330, 331]. Spatial epigenomic approaches map chromatin states to tissue microenvironments in situ [344]. Locus‐resolved perturbation and epigenome editing, including CRISPR‐dCas9 effector fusions, allow direct tests of candidate regulatory elements and mark dependence [284, 285].

Translation depends on alignment between the mechanism, cellular compartment, and pharmacology, while recognizing that mechanistic evidence is predominantly preclinical. For drug‐like approaches, target engagement and modulation of the intended histone mark should be demonstrated in cardiovascular tissues, and dose selection should be guided by quantitative PD readouts rather than downstream phenotypes alone. Metabolic interventions require matched quantification of relevant metabolite abundance and site‐resolved PTM changes within the same compartment [158, 275], because pathway signatures can be misleading when PTM function is cell‐state dependent. Human tissue‐based epigenomic resources, including cardiac H3K27ac maps, link regulatory element activity to remodeling programs [311] and enable mechanistic prioritization of noncoding risk loci [310]. However, access to human myocardial tissue remains constrained and is typically limited to intraoperative biopsies, explants, autopsy material, or unused donor hearts rather than routine longitudinal sampling [310, 311]. What now limits translation is not the cataloguing of histone PTMs itself, but the ability to assign targetable chromatin mechanisms to the relevant cell state, genomic locus, and stage of disease. Progress toward cardiovascular epigenetic therapeutics will therefore require human‐guided target prioritization, direct validation of target–mark–phenotype relationships, and PD readouts that demonstrate on‐target chromatin modulation in vivo.

## Author Contributions

Yu Zheng and Yu‐Xuan Gao wrote the manuscript and produced the figures. Mei‐Xing Guo, Li‐Ting Wang, Xiao‐Wen Zheng, and Yi‐Hao Liao reviewed the manuscript and contributed to the revision. Ya Li, Jun‐Ping Zhu, and Jia‐Ming Wei coordinated the work and led the revision of the manuscript. All authors contributed to the manuscript and approved the version submitted.

## Funding

This work was supported by the National Natural Science Foundation of China (No. 82405372), the Postdoctoral Fellowship Program of CPSF (GZC20252636), the China Postdoctoral Science Foundation (2025M773968, 2025T181082), Changsha Science and Technology Bureau Project (kq2402175), 2024 National Innovation and Entrepreneurship Training Program for College Students of Hunan University of Chinese Medicine (S202410541016), the Scientific research project of Hunan University of Chinese Medicine (Z2023XJYB05), and Undergraduate Innovation Project of Hunan University of Chinese Medicine (2023BKS021, 2024BKS146).

## Ethics Statement

The authors have nothing to report.

## Conflicts of Interest

The authors declare no conflict of interest.

## Data Availability

The authors have nothing to report.
